# Decoding the Functional Interactome of Non-Model Organisms with PHILHARMONIC

**DOI:** 10.1101/2024.10.25.620267

**Published:** 2024-10-29

**Authors:** Samuel Sledzieski, Charlotte Versavel, Rohit Singh, Faith Ocitti, Kapil Devkota, Lokender Kumar, Polina Shpilker, Liza Roger, Jinkyu Yang, Nastassja Lewinski, Hollie Putnam, Bonnie Berger, Judith Klein-Seetharaman, Lenore Cowen

**Affiliations:** * Center for Computational Biology, Flatiron Institute, New York, NY, USA; † Department of Computer Science, Tufts University, Medford MA, USA; ‡ Departments of Biostatistics & Bioinformatics and Cell Biology, Duke University, Durham, NC, USA; § Shoolini University, Solan, Himachal Pradesh-173229India; ¶ School of Molecular Sciences, Arizona State University, Phoenix, AZ, USA; ‖ Department of Mechanical Engineering, Seoul National University, Seoul, South Korea; ** Department of Chemical and Life Science Engineering, Virginia Commonwealth University, Richmond, VA, USA; †† Department of Biological Sciences, University of Rhode Island, Kingston, RI, USA; ‡‡ Computer Science & Artificial Intelligence Laboratory and Department of Mathematics, MIT Cambridge, MA, USA

## Abstract

Protein-protein interaction (PPI) networks are a fundamental resource for modeling cellular and molecular function, and a large and sophisticated toolbox has been developed to leverage their structure and topological organization to predict the functional roles of under-studied genes, proteins, and pathways. However, the overwhelming majority of experimentally-determined interactions from which such networks are constructed come from a small number of well-studied model organisms. Indeed, most species lack even a single experimentally-determined interaction in these databases, much less a network to enable the analysis of cellular function, and methods for computational PPI prediction are too noisy to apply directly. We introduce PHILHARMONIC, a novel computational approach that couples deep learning *de novo* network inference with robust unsupervised spectral clustering algorithms to uncover functional relationships and high-level organization in non-model organisms. Our clustering approach allows us to de-noise the predicted network, producing highly informative functional modules. We also develop a novel algorithm called ReCIPE, which aims to reconnect disconnected clusters, increasing functional enrichment and biological interpretability. We perform remote homology-based functional annotation by leveraging hmmscan and GODomainMiner to assign initial functions to proteins at large evolutionary distances. Our clusters enable us to newly assign functions to uncharacterized proteins through “function by association.” We demonstrate the ability of PHILHARMONIC to recover clusters with significant functional coherence in the reef-building coral *P. damicornis*, its algal symbiont *C. goreaui*, and the well-annotated fruit fly *D. melanogaster*. We perform a deeper analysis of the *P. damicornis* network, where we show that PHILHARMONIC clusters correlate strongly with gene co-expression and investigate several clusters that participate in temperature regulation in the coral, including the first putative functional annotation of several previously uncharacterized proteins. Easy to run end-to-end and requiring only a sequenced proteome, PHILHARMONIC is an engine for biological hypothesis generation and discovery in non-model organisms.

PHILHARMONIC is available at https://github.com/samsledje/philharmonic

## 1 Introduction

There exists a rich ecosystem of methods that leverage known protein-protein interaction (PPI) networks to cluster genes and proteins into functionally-enriched modules [17, 54, 72], or that use network-propagation to directly infer protein labels based on the graph-theoretic structure [20]. PPI databases such as STRING [79] and BioGRID [62] play a crucial role by aggregating experimentally determined PPIs into networks, which then enable the deployment of these powerful tools for functional annotation. However, an overwhelming proportion of known PPIs in such databases are from humans and a small number of well-studied model organisms (Section A.1), and for the vast majority of non-model organisms experimentally determined PPI interactions are too rare to establish a useful network for these types of inference.

There is likewise no existing computational method that can infer a sufficiently useful network. It is well-known that even over relatively short evolutionary distances there is substantial network re-wiring [7], so that even when sequence and protein structure conservation can allow confident ortholog assignment from a well-studied species, the interaction patterns are often not preserved. Even at a moderate evolutionary distance, the presence of an interaction can differ between two species, and the probability of shared edges decreases as the evolutionary distance between two species increases—so the networks from well-studied organisms such as human cannot trivially be used to transfer interactions to non-model organisms. Existing deep learning methods that predict PPIs are either too slow to make genome-wide predictions [27, 4, 90, 43] or are not accurate enough to use the raw predicted network as ground truth interactions [16, 74, 80].

Here, we introduce **PHILHARMONIC** (Protein Human-Transferred Interactome Learns Homology And Recapitulates Model Organism Network Interaction Clusters), a novel bioinformatics framework for network, community, and functional inference in non-model organisms. We show that PHILHARMONIC makes meaningful predictions of PPI and protein function when applied to coral, algae, and fruit fly proteomes. The key insight behind PHILHARMONIC is that PPI networks predicted by high-throughput computational methods, while not at the level of experimental correctness, nonetheless contain substantial signal of functional relatedness. **With innovative downstream processing, these *de novo* networks can serve as an information-rich scaffold from which to perform pathway-level network analysis and extract functional genomics.**

**Figure 1:**
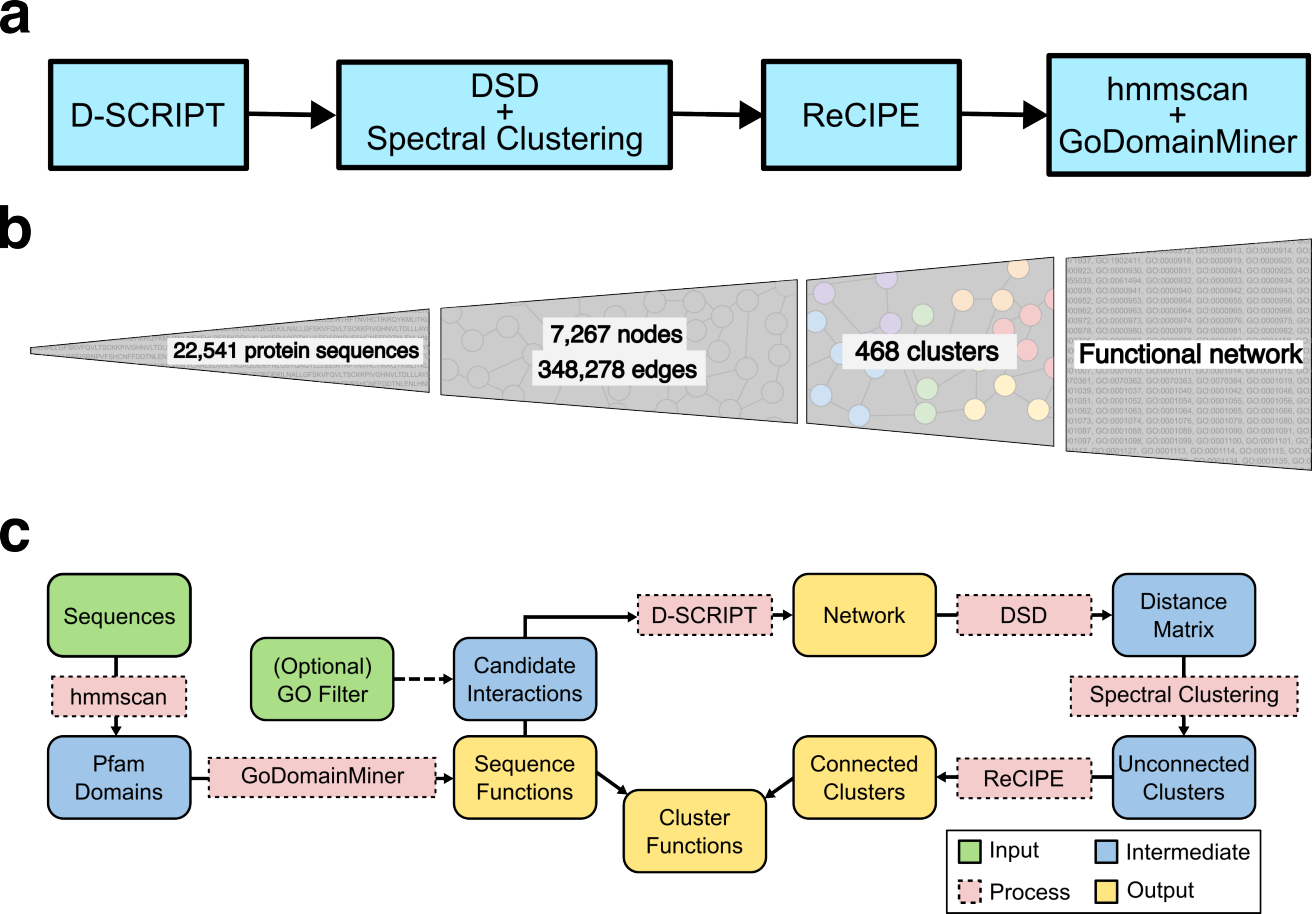
PHILHARMONIC is an engine for biological discovery in non-model organisms. **(a)** High-level overview of PHILHARMONIC. We use D-SCRIPT [75] to predict a *de novo* protein-protein interaction network (optionally on a smaller set of GO-filtered proteins, if computational cost is an issue), then cluster this network using DSD [12], recursive spectral clustering [60], and ReCIPE (see Figure 3). We use hmmscan [25] and GoDomainMiner [1] to map sequence functions, then use the individual sequence functions to assign cluster functions, which allows for network-level discovery and hypothesis generation. **(b)** PHILHARMONIC requires only a sequenced proteome, which provides on its own little functional information. We integrate several other sources of information and downstream processing to build up to a functional network. **(c)** More detailed diagram of the PHILHARMONIC method, where we show individual inputs, outputs, intermediate data, and the tools we use to transform them, including the newly-developed ReCIPE.

PHILHARMONIC has four primary stages (Figure 1a). First, we use the deep learning method D-SCRIPT (a deep learning PPI prediction method shown to have state-of-the-art performance [76] in non-model organisms) to infer the initial, noisy PPI network. We then build a novel recursive clustering algorithm based on the Double Spectral method, which couples a Diffusion State Distance (DSD)-based similarity metric [13] with several rounds of spectral clustering [60] to convert the PPI network into putative functionally-enriched non-overlapping clusters of balanced sizes amenable to easy biological analysis. These clusters are by definition non-overlapping and can be highly disconnected, as opposed to in biology where proteins can play many functional roles. To rectify this, we develop a novel method, ReCIPE (Reconnecting Clusters In Protein Embedding), a greedy algorithm that optimizes intra-cluster connectivity by re-adding high degree nodes and creates coherent, better connected, and explainable clusters. We employ remote homology methods that give sequence-based gene ontology (GO) annotations to proteins based on Pfam domains [26], and our clusters inherit the functions that are enriched in its constituent proteins. On a gene-by-gene basis, we have previously demonstrated that some functional inference can still be accomplished by remote homology approaches using profile-profile HMMs [47]. We demonstrate here that when combined with functionally-enriched clusters, these annotations can be successfully transferred to non-annotated proteins to infer larger functional pathways. Finally, we use a generative AI tool to summarize the function of each cluster in paragraph form, providing easily readable summaries of cluster functions for the user.

Corals are important natural resources that are key to the ocean’s vast biodiversity and provide economic, cultural, and scientific benefits [9, 11, 66]. While they cover only 0.1% of the ocean floor, coral reefs are home to the largest density of animals on earth, rivaling rain forest habitats in species diversity [34]. As a result of anthropogenic activities, coral holobionts are declining rapidly (Section A.2). This trajectory of loss highlights the extreme urgency to assist corals in overcoming the impacts of pollution and global warming before the damage to these vital reef ecosystems is irreparable. New efforts are underway to harness the growing amount of genomic information that is becoming available for the coral animal, its hosted symbiotic algae, and other elements of the coral holobiont, to discover ameliorative strategies that could slow coral decline [21]. However, these efforts are hampered by the great evolutionary distance of corals to any species with functionally well-annotated genes and genomes.

The importance of corals led us to apply PHILHARMONIC to the proteome of the coral *Pocillopora damicornis*, [22] (Section 3.4) and to one symbiont species, *Cladocopium goreaui* [55] (Section A.7), newly assigning predicted functional labels to hundreds of un-annotated proteins. We spotlight two clusters enriched with functions important for coral resilience, temperature sensing (Section 3.4.1) and response to environmental stimuli (Section 3.4.2). These clusters include proteins that homology methods fail to annotate, shedding some light on the so-called “dark proteome” [78] of proteins that are too evolutionary distant from model organisms for remote homology methods to predict their functions. We support these results by showing significant functional coherence in our clusters with both GO annotations (Section 3.1) and gene co-expression (Section 3.2) from Connelly et al. [19]. We perform a similar analysis on *C. goreaui* in Section A.7. As orthogonal validation of our method, we evaluate ReCIPE on recent DREAM Challenge networks (Section 3.3) where we demonstrate that the reconnection step yields more functionally enriched clusters. We also run PHILHARMONIC on the fruit fly proteome (*Drosophila melanogaster*), where we show significant enrichment for canonical GO functions from FlyBase [83] (Section A.8). Our work demonstrates the ability of methods like PHILHARMONIC to realize the promise of systems biology, particularly with respect to understanding non-model organisms.

## 2 Methods

### 2.1 Data Collection

In this work, we focus on the coral *Pocillopora damicornis* and its dinoflagellate symbiont *Cladocopium goreaui* (formerly named *Symbiodinium goreaui*, Clade C type C1). Coral protein sequences were obtained from Cunning et al. [22], while symbiont sequences were obtained from ReefGenomics [53, 88], based on Liu et al. [55]. After filtering to proteins with between 50 and 800 residues due GPU VRAM limitations, we were left with 22,541 *P. damicornis* sequences and 28,271 *C. goreaui* sequences. We also perform analysis on the fly *Drosophila melanogaster* genome, using sequences from FlyBase [83]. We keep 11,487 fly sequences with between 50 and 800 residues.

### 2.2 Functional Annotation of Individual Genes

Remote homology methods can find related genes that might preserve functional roles over greater evolutionary distance than standard sequence-based or ordinary-HMM-based searches. We employ a two-step functional annotation to maximize conserved function. We construct an HMM database from Pfam [26] domains using hmmer [25], then use hmmscan to search our input sequences against this database. Each protein is then annotated with some number of domains. Finally, we use mappings between domains and GO biological process (GO:BP) functions from GODomainMiner [1] to transfer functional terms to candidate sequences. Because structured domains are more likely to be conserved across long evolutionary distance, this procedure ensures the highest quality functional annotations for single proteins—but still results in some proteins which are unable to be functionally characterized, or may yield false positive annotations.

### 2.3 Synthetic Network Prediction

D-SCRIPT [75] is a deep learning method that was trained on human PPIs and has been shown to generalize broadly across species. It requires only the amino acid sequences of two proteins and predicts a probability of interaction. D-SCRIPT can either be run on all pairs of proteins, or, if there are computational constraints, instead the set of proteins can be curated based on a list of GO terms. In our case, we use a hand-curated list of diverse high-level GO:BP terms potentially related to coral metabolism, resilience, and regulation (see Section A.4 for this full list). We keep any proteins which are annotated with these high-level GO terms or any child GO terms. We generate a list of candidate interactions by considering all pairwise interactions between the filtered set of proteins, resulting in 45.9 million coral candidates, 52.7 million symbiont candidates, and 15.2 million fly candidates. We use D-SCRIPT because it is both highly accurate for non-model organisms and computationally efficient enough to be run genome-wide on the proteins in a new organism—it takes approximately a week to predict ≈ 50 million interactions on a single GPU. We consider a positive interaction to be anything predicted with a probability greater than 0.5.

### 2.4 Diffusion State Distance

DSD is an effective way to measure distance between nodes in a PPI network. We first consider it in the case the edges are unweighted as in [13]. For two nodes (proteins) a and b, we measure the expected number of times a *k*-step random walk starting at a visits b (including the 0-step random walk), and call this $He^{k}(a,b)$. If $v_{1},\ldots v_{n}$ represent all the nodes in the network, we then associate to each node $v_{i}$ the vector consisting of these $He^{k}$ values to all n nodes, namely

(1) $$He^{k}\left(v_{i}\right)=\left(He^{k}\left(v_{i},v_{1}\right),He^{k}\left(v_{i},v_{2}\right),\ldots He^{k}\left(v_{i},v_{n}\right)\right)$$

Then the DSD distance between two nodes $v_{i}$ and $v_{j}$ is defined as the $L_{1}$ norm of the vectors $He^{k}\left(v_{i}\right)$ and $He^{k}\left(v_{j}\right)$, i.e.

(2) $$DSD^{k}\left(v_{i},v_{j}\right)={\left\|He^{k}\left(v_{i}\right)-He^{k}\left(v_{j}\right)\right\|}_{1}$$

Cao et al. (2013) [13] show that this converges (rapidly, in small world networks) as the length of the random walk k→∞, and denote the $DSD\left(v_{i},v_{j}\right)$ without the k superscript to mean the converged DSD. This can be computed exactly with the closed form in [13]. Furthermore, when there are weights on the edges, a straightforward modification presented in Cao et al. (2014) [12] can take into account confidence weights on the edges by instead biasing the underlying random walk to more likely go along edges of more confidence (in direct proportion to the confidence ratios among the edges from the node). Everything else in the weighted DSD definition stays the same. We set the confidences according to the edge weights predicted by D-SCRIPT.

### 2.5 Community Detection

Given the DSD-smoothed node distance matrices, we convert this to a similarity measure between nodes using the RBF kernel [38]. We cluster this distance matrix using a modified version of the spectral clustering algorithm described in [60], recursively splitting any cluster that was bigger than assigned threshold. This DSD + spectral clustering method won the disease module identification challenge in DREAM 2016 [17]. Our version of this algorithm requires three parameters K,D,M. For the default PHILHARMONIC implementation, we initially call spectral clustering with K=500 clusters. The initial set of clusters is recursively split, where clusters are recursively divided into $round\left(\frac{n}{D}\right)$ clusters (where n is the size of the cluster) until $round\left(\frac{n}{D}\right)<2$. Since the network will display cluster organization at multiple scales, we chose the default D=20 to create clusters of a size reasonable for biologists to further investigate by hand (resulting in a maximum cluster size of (1.5×D)−1=29). We finally discard clusters with fewer than M=3 nodes.

### 2.6 Reconnecting Clusters

We observed that because DSD groups together nodes that are most similar, these clusters often placed nodes in the same cluster because they were all connected to the same intermediate-degree minor hub nodes, but these hub nodes were instead grouped with other minor hub nodes, producing highly disconnected clusters. We develop a new method, ReCIPE (Reconnecting Clusters in Protein Embeddings) to return the minor hubs back into these clusters if they were otherwise disconnected, allowing some overlap between clusters (Figure 3). Proteins were considered for re-inclusion in the cluster (“candidate proteins”) if they connected at least 10% of the disconnected cluster components. Then the candidate proteins are added back in increasing order of degree. If n is the number of proteins in the cluster, and c is the number of disjoint cluster components, then proteins are added until the connectivity score $s_{c}=1-\frac{\left(c-1\right)}{\left(n-1\right)}>0.75$, up to a maximum of 20 re-added proteins (where we note that a fully connected cluster has a $s_{c}$ of 1, and that since clusters contain at least 3 nodes, the denominator is always defined (and at least 2)). We selected these parameters from independent experiments on human PPI networks derived from the DREAM challenge [17], where they led to clusters that were better connected and more functionally enriched than the original (Section A.9). We also show that the ReCIPE method does not result in clusters that are massively overlapping and that even after the ReCIPE reconnection step, most nodes are not placed in too many clusters (Figure A13).

### 2.7 Creating the Cluster Graph

To aid in the analysis of functional clustering in the network, we compute a higher-order graph where each node is not a protein, but rather a PHILHARMONIC-determined cluster. Given cluster C with nodes $\left\{c_{1},\ldots ,c_{n}\right\}$, cluster D with nodes $\left\{d_{1},\ldots ,d_{m}\right\}$, and original adjacency matrix $A\in {\left\{0,1\right\}}^{N\times N},\;N>n,m$, we construct a new graph G with edges $(C,D)=\sum_{c_{i},d_{i}}A_{c_{i},d_{i}}$. Finally, we drop all edges with weight less than t=10 higher levels of t for visualization. When constructing this graph, we do not consider ReCIPE-added nodes in the edge count, as ReCIPE results in potentially overlapping clusters, leading to potentially double-counting edges if the same node appears in both C and D. We assign each cluster node a single function, based on GO Slim [3] terms appearing most frequently within that cluster. A given GO term must appear in at least 3 proteins in the cluster to be assigned, otherwise the cluster is left with an unassigned function.

### 2.8 LLM Summarization

Inspired by work from Hu et al. [35], we sought to improve the interpretability of our clusters by using large language models (LLMs) to automatically annotate them with names. Not only does this more easily summarize cluster function, but we found that it eases communication within teams of users when referring to clusters. We utilize the llm command line utility [89], prompting the GPT 4o-mini model with a short text summary of each cluster containing the size, number of edges/triangles, and the top-represented GO terms within each cluster. The model is instructed to act as an expert biologist, and to come up with a short, descriptive, human-readable name for each cluster. The full system prompt is include in Section A.5.1. This name is added to the text summary from above and output for biological analysis (Figures 5a, 6a). We note that, similarly to Hu et al., the correctness of cluster labels will be highly dependent on the prompt and the choice of LLM. These cluster names should be used as a helpful supplement rather than an absolute statement of truth about the function of the cluster.

### 2.9 Implementation

We implement PHILHARMONIC using Snakemake [58]. This allows for consistent and reproducible analysis, flexible configuration with a .yaml file, and flexible allocation of computational resources. We use HMMER v3.3.0, D-SCRIPT v0.2.8, and fastDSD v1.0.1. ReCIPE is a newly implemented and pip-installable Python package; we use v0.0.4 in this work. Each clustering and analysis step is implemented as a Python script which is run by Snakemake, and can be individually called through the PHILHARMONIC package. We use the spectral clustering implementation from scikit-learn version 1.5.0. Statistical tests were performed with scipy version 1.11.3. We use Python version 3.11 and Snakemake version 8.10. D-SCRIPT was run on a single A6000 NVIDIA GPU, and hmmscan was run using 32 cores. Cytoscape 3.10.2 was used for visualization of all networks, both protein-protein interaction and higher level cluster graphs. We provide a set of Cytoscape style files that can be used to visualize assigned cluster functions. PHILHARMONIC is available at https://github.com/samsledje/philharmonic. ReCIPE is available at https://github.com/focitti/ReCIPE.

## 3 Results

### 3.1 PHILHARMONIC clusters are functionally coherent

**Figure 2:**
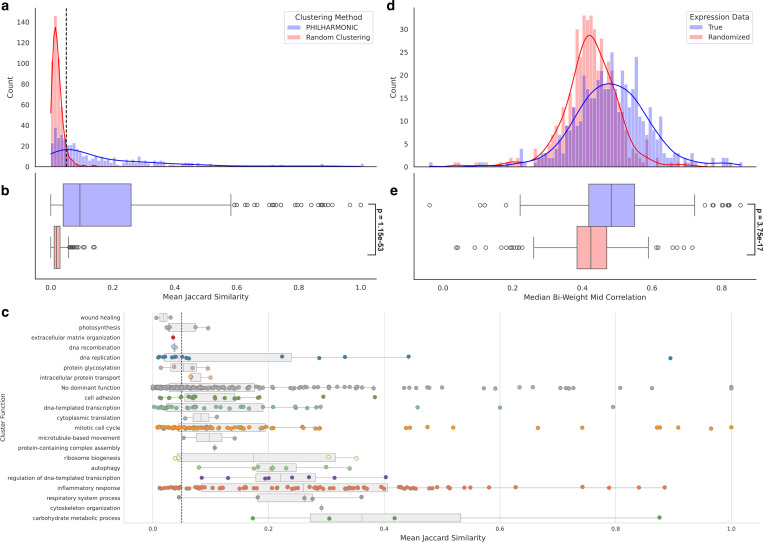
Validation of PHILHARMONIC clusters in *P. damicornis*. **(a,b)** We compute a cluster coherence as the mean Jaccard similarity between the sets of GO functional terms assigned to each pair of proteins in the same cluster. Compared to a distribution-preserving random clustering of the network, PHILHARMONIC clusters are significantly more functionally coherent (*p* = 1.15 × 10^−53^ one-tailed independent t-test). We use this test to set a cluster coherence threshold of 0.05 (dashed grey line) where the two curves cross, above which nearly every cluster is unlikely to be drawn from the random distribution. **(c)** We show each of the 486 clusters, separated by GO Slim functional assignment and plotted by cluster coherence. We find a substantial number of clusters above the 0.05 threshold across several diverse functions, especially mitotic cell cycle (GO:0000278), DNA-templated transcription (GO:0006351), and inflammatory response (GO:0006954). We show the same threshold at 0.05. Points are colored by cluster functions from Figure 4c. **(d,e)** Using *Pocillopora* gene expression data from Connelly et al. [19], we compute the median bi-weight mid correlation between pairs of genes in the same cluster, and compare this to shuffled expression data. We find that proteins that share a PHILHARMONIC cluster are significantly more likely to be co-expressed (*p* = 3.75 *×* 10^−17^, one-tailed relative t-test).

We set out to determine whether PHILHARMONIC generated functionally meaningful clusters by investigating the level of functional coherence within a cluster. We define functional similarity between two proteins as the Jaccard similarity between the sets of GO [3] terms assigned to each protein by our Pfam-based function annotation. Then, the cluster coherence is the mean functional similarity of all pairs of proteins in the cluster. We compare PHILHARMONIC clusters with a degree-preserving random clustering, constructed by randomly shuffling node function assignments. We find that *P. damicornis* clusters computed by PHILHARMONIC conserve significantly more functional relationships (*p* = 1.15 × 10^−53^, one-tailed independent samples t-test) (Figure 2a). Using the most-represented GO Slim term assigned to each cluster (Section 2.7), we show the cluster coherence of clusters with each high-level function in Figure 2c. We find several highly-coherent clusters related to mitotic cell cycle (GO:0000278), DNA-templated transcription (GO:0006351), and inflammatory response (GO:0006954). We perform the same analysis using only GO Slim [3] terms in Section A.6.1. We replicate this analysis on the *C. goreaui* network (Section A.7.1), and the *D. melanogaster* network using gold-standard GO terms from FlyBase [83] (Section A.8).

### 3.2 PHILHARMONIC clusters correlate with gene expression

We likewise investigated whether the PHILHARMONIC *P. damicornis* clusters are more likely to group proteins which are co-expressed. We use gene expression data from Connelly et al. [19], consisting of 47 samples of RNAseq data from four *Pocillopora* coral specimens, collected in triplicate across four conditions (control, heat exposure, antibiotics exposure, heat+antibiotic exposure). Following [19], we compute gene co-expression scores by first computing a variance-stabilizing transform on expression values, then computing the bi-weighted mid-correlation statistic between genes. Genes with significant missing data (0 counts in $>\frac{1}{2}$ of samples) were removed. As a baseline, we re-shuffle expression, preserving the distribution of expression values. We find that proteins in PHILHARMONIC clusters are significantly more likely to be co-expressed across these tested conditions ($p=1.27\times 10^{-21}$, one-tailed related samples t-test) (Figure 2d,e).

### 3.3 Reconnecting clusters improves functional enrichment

**Figure 3:**
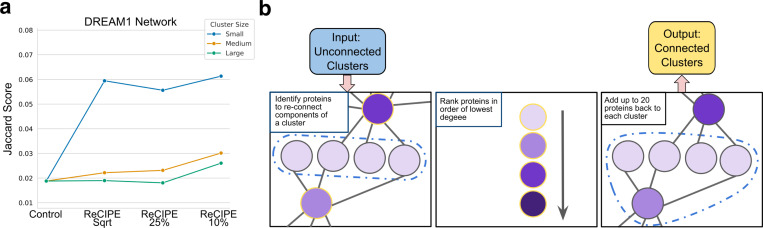
Reconnection with ReCIPE results in more functionally enriched clusters. **(a)** Jaccard score of function predictions for DREAM1 network when clusters are computed using ReCIPE at different linear ratio levels, compared to a control of disconnected clusters. We separate clusters into small (8–16 nodes), medium (17–32), and large (33–64) to better visualize performance. Generally, ReCIPE has the large impact on small clusters, likely because functionally informative hub nodes are preferentially placed in large clusters by the spectral algorithm. We find that a linear ratio of 10% yields the overall best performance, and thus use this setting for our coral experiments. We show a similar analysis using the percentage of enriched clusters, and on DREAM2 and DREAM3, in Section A.9. **(b)** Schematic overview of the ReCIPE method. ReCIPE identifies candidate nodes for re-addition, ranks them prioritizing low-degree nodes, and adds them back greedily until a connectivity threshold or maximum number of proteins is hit.

We apply ReCIPE to three human PPI networks with known functions sourced from the Dialogue on Reverse Engineering Assessment and Methods (DREAM) challenge [17] (Section A.9 for further network details). We compute the set of enriched GO terms for a cluster using v2.0.1 of the FUNC-E Python package [28]. We then assign GO functions based on the enriched terms, and compute a Jaccard similarity between the predicted and true sets of functional labels. We show in Figure 3 and Figure A12 that ReCIPE clusters generally perform at least as well as non-overlapping clusters for all three DREAM networks, and are often much better for functional annotation in addition to their improved interpretability. ReCIPE with a linear ratio of 10% overall performs the best, so we select this linear ratio for our analysis of the *P. damicornis*, *C. goreaui*, and *D. melanogaster* networks. We perform a similar analysis where we score function predictions not by Jaccard score, but by an F1 score computed over the top 10 most strongly enriched functions (by *p*-value) in Section A.9.

### 3.4 Dissecting the *P. damicornis* network

**Figure 4:**
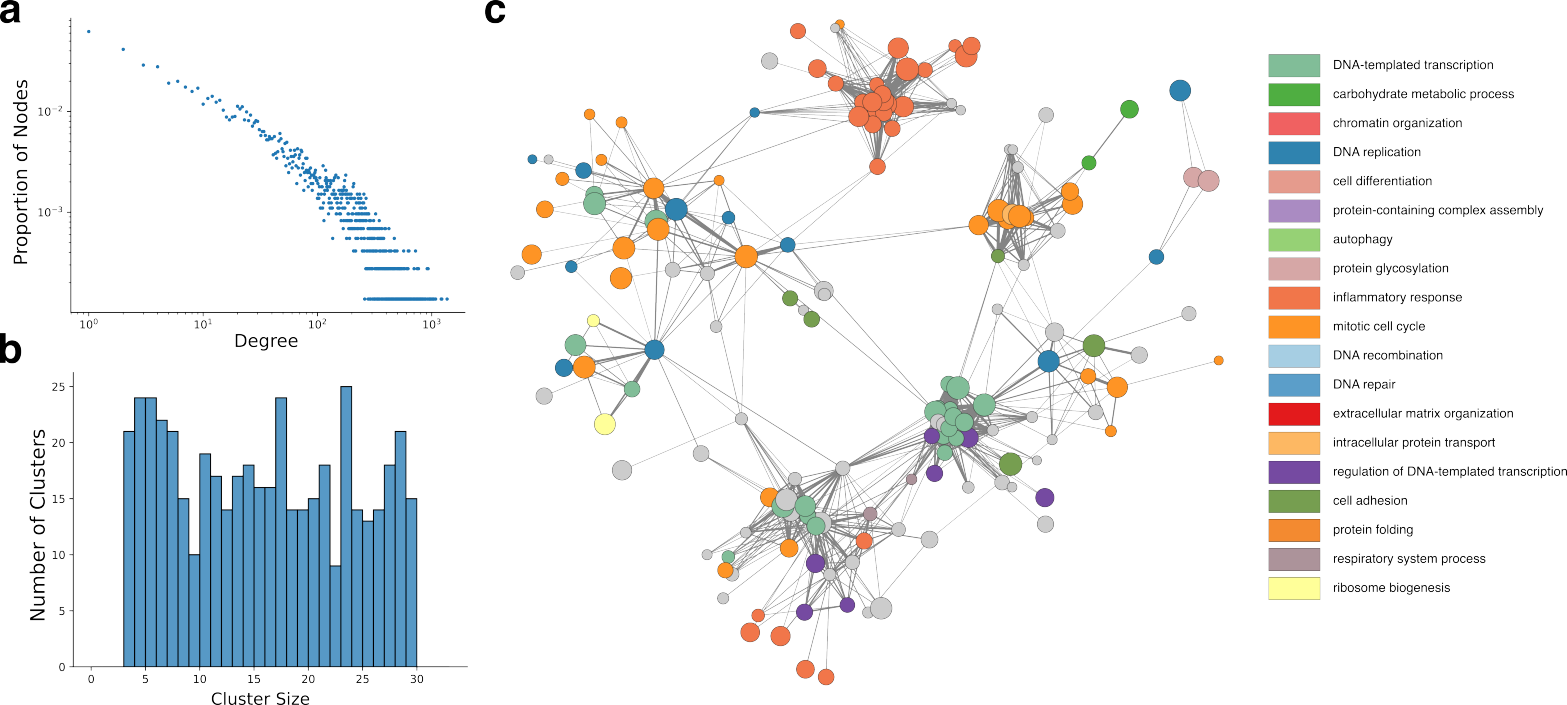
Dissecting the functional network of the coral *P. damicornis*. **(a)** The synthetic PPI network predicted by PHILHARMONIC follows a scale-free degree distribution commonly found in biological networks [2]. **(b)** We perform DSD followed by a hierarchical spectral clustering on the network, yielding clusters with sizes between three and 29 nodes. **(c)** The *P. damicornis* cluster graph, where each node is a cluster and edges indicate high cross-connectivity between clusters. Each cluster is colored by the predominant high-level function of constituent proteins. We find neighborhoods of the network focused on inflammatory response (GO:0006954, dark orange), mitotic cell cycle (GO:0000278, light orange), and DNA-templated transcription (GO:0006351, green) and its regulation (GO:0006355, purple).

Reef building coral play a crucial role in the reef ecosystem. We therefore chose to apply PHILHARMONIC on a case study in the coral *P. damicornis* (We also apply PHILHARMONIC to *C. goreaui* in Section A.7) and *D. melanogaster* in Section A.8). The *P. damicornis* network displays scale-free characteristics [2] typical of biological networks (Figure 4a), and the distribution of cluster sizes is balanced and intentionally constrained by our recursive splitting procedure (Figure 4b). Further network statistics can be found in Table A2. Additionally, we show the high-level functional organization of the network where each node is itself a cluster (Figure 4c, see Section 2.7 for details). We find substantial organization within this network, with different functions partitioned to different “neighborhoods” of the network. This view of the network highlights the centrality of gene regulation and the cell cycle in cellular function [67], and we identify a tightly-connected neighborhood of the overall network dedicated to inflammatory response [84].

In addition to this general view, we investigate two clusters in depth, both of which involve ion channels communicating with G-coupled protein receptors (GPCRs). A large repertoire of GPCRs with homology to neuropeptide receptors that modify behavior in the vertebrate brain seem to have ancient origin, and predate the invertebrate-vertebrate split, and thus can be found in cnidaria such as hydra and coral animals [64], despite their lack of a traditional brain organ. The pathways involving these genes form a central nervous system in coral polyps and could effect coral behavior and plasticity to adapt to, e.g. heat stress. Indeed, we demonstrate heat-stress differential expression in multiple genes in these clusters using data from an independent study [19]. Using these clusters, we perform novel functional analysis of previously uncharacterized proteins.

#### 3.4.1 Cluster involved in regulation of thermal response in *P. damicornis*

**Figure 5:**
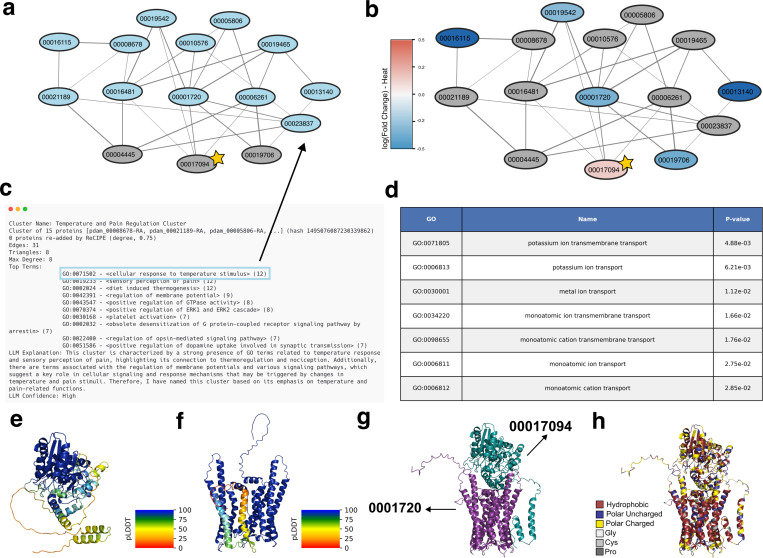
Example cluster involved in temperature and pain regulation in *P. damicornis*. **(a)** Graph of this cluster, where proteins annotated with cellular response to temperature stimulus (GO:0071502) are highlighted in blue. **(b)** Colored by *log*_2_(Fold change) in expression when exposed to heat [19] (genes not in data are colored gray). **(c)** Human-readable PHILHARMONIC output for selected cluster. **(d)** Enriched GO terms identified by g:Profiler [45] **(e)** AlphaFold-predicted [40] structure of pdam 00017094, an uncharacterized protein (starred above), colored by pLDDT. **(f)** AlphaFold-predicted structure of pdam 0001720, a putative allatostatin-A receptor, colored by pLDDT. **(g)** AlphaFold-Multimer-predicted [27] structure of their interaction. **(h)** Colored by amino acid type, highlighting the likely membrane association of the complex.

We analyze here one PHILHARMONIC cluster involved in cellular response to temperature stimulus (GO:0071502) (Figure 5). Cnidarians, including coral animals, have a large and diverse family of voltage-gated K+ channels, but little is known about how the function of these channels differs across different cnidarian species [49]. This cluster includes three proteins that are likely voltage-gated K+ channels (pdam 00019542, pdam 00019465, pdam 00010576), a cyclic nucleotide-gated K+ channel (pdam 0008678, [56]), and an HCN channel (pdam 00005806). HCN channels are involved in controlling neuronal excitability, dendritic integration of synaptic potentials, synaptic transmission, and rhythmic oscillatory activity in individual neurons and neuronal networks in humans, where they have been studied for their role in epilepsy and pain [8]. Cnidarian HCN channels share all major functional features of vertebrate HCNs, including reversed voltage-dependence, activation by cAMP and PIP2, and blocking by extracellular Cs+ [5]. There is some evidence that HCN channels are also involved in rhythmic firing and the so-called pacemaker channels [71]. This cluster also contains several GPCRs that are predicted to bind to and likely modulate the functions of the above diverse ion channels in response to different stimuli. Notably, many of these receptors are linked to neural peptides (Section A.6.2), including an orexin receptor (pdam 00006261) that has been implicated in systems involving sleep and light-sensing in mammals [42]. pdam 00001720 resembles the allatostatin-A receptor, to which the neuropeptide allatostatin-A binds, modulating feeding behavior in mosquitos [18].

We identify one protein, predicted to bind to a subset of the GPCRs, including the putative orexin and allatostatin-A receptors, which is completely uncharacterized (pdam 00017094, starred in Figure 5b and c). Not only were we unable to identify homologs through sequence similarity search, but we also could not find structural homologs using FoldSeek [86]. Using data from Connelly et al. [19], we find that 00017094 is slightly up-regulated during heat stress. We use AlphaFold [40, 27] to predict the structure of 00017094 in complex with 0001720, as well as their individual structures. 00017094 contains two likely flexible helices, predicted with low plDDT (Figure 5e), that are predicted to sit alongside the barrel in the complex structure (Figure 5h). Due to the number of polar residues in these helices, it is more likely that these helices float on the surface of the membrane, or interact with another protein. These structures support the conclusion that 0001720 is likely a membrane-associated receptor, and suggest that 00017094 may bind on the outside of the receptor. It is tempting to speculate that its binding may regulate the allatostatin-A receptor and coral feeding behavior as a response to heat stress.

#### 3.4.2 Cluster involved in neurophysiological response to environmental stimuli in *P. damicornis*

**Figure 6:**
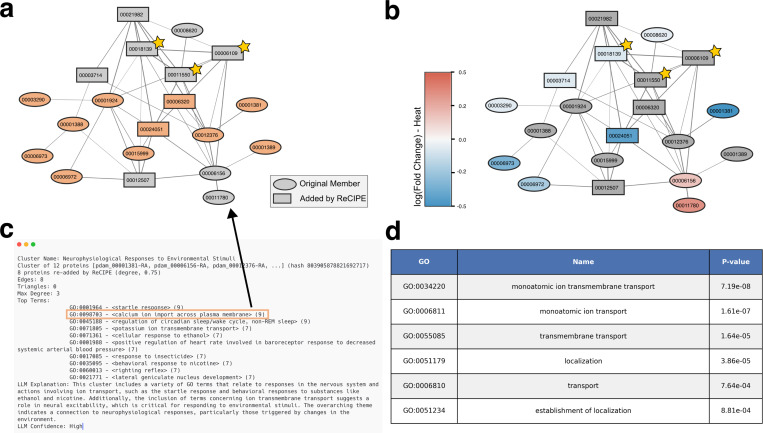
Example cluster involved in neurophysiological response to environmental stimuli in *P. damicornis*. **(a)** Graph of this cluster, where proteins annotated with calcium ion import across the plasma membrane (GO:0098703) are highlighted in orange. **(b)** Colored by *log*_2_(Fold change) in expression when exposed to heat [19] (genes not in data are colored gray). Previously uncharacterized proteins are annotated with gold stars. **(c)** Human-readable PHILHARMONIC output for selected cluster. **(d)** Enriched GO terms identified by g:Profiler [45]

This cluster likewise contains two proteins with high levels of homology to K+ voltage-gated channels [65], namely pdam 00001381 in the original cluster and pdam 00006320 among the proteins added in by ReCIPE. Among the remaining proteins include several highly homologous to ligand-gated ion channels that have been recognized as key modifiers of behavior in vertebrate brains, four gamma-aminobutyric acid (GABA) receptors (pdam 00012376-RA, pdam 00001388, pdam 00001389 and pdam 00024051, the last added by ReCIPE), and four that are likely nicotinic receptors (pdam 00006972, pdam 00015999, pdam 00006973, pdam 00012507). GABA has been identified as a signalling molecule in feeding behaviour, orientation, and tentacle movement in other Cnidarian species [77]. Jing et al. [39] showed that the roles of GABAergic inhibitory interneurons can also be extended to the Aplysia feeding motor network. They are widely expressed in the central nervous system, while, in the periphery, they mediate synaptic transmission at the neuromuscular junction and ganglia. There are three proteins in this cluster which have previously been uncharacterized – pdam 00011550, pdam 00018139, and pdam 00006109, which we highlight in Figure 6 with stars. Two of these, 00011550 and 00006109, are likely interacting with a GABA receptor candidate 00024051 that is highly down-regulated in heat.

## 4 Discussion

While substantial previous work has advanced our understanding of functional networks in humans [29, 15, 30, 44, 37], limited experimental data and large evolutionary distance from well-studied organisms has made the analysis of functional networks in non-model organisms difficult. Methods for remote homology detection [47] or structure-based search [87] are important steps in this direction, but network re-wiring between species [73] limits their applicability to genome-scale pathway analyses. Recently developed machine learning methods to transfer PPIs from humans to other species allows the generation of useful networks [75], but standalone these networks are too noisy to apply directly to function prediction. To address these limitations, we have developed PHILHARMONIC. Our approach combines protein language models, spectral algorithms, and traditional HMM-based sequence algorithms to generate functionally meaningful and biologically interpretable clusters. We validate the approach using the literature, gene expression data, and we couple our methods with protein 3D structure predictions to investigate several previously uncharacterized proteins related to thermal and environmental response in the coral *P. damicornis* that it would not be possible to annotate based on sequence or structural information alone. We show that our results extend beyond a single species by applying PHILHARMONIC to the algal symbiont *C. goreaui* and the well-annotated fruit fly *D. melanogaster*.

There still remain several challenges and opportunities to improve the understanding of functional networks in non-model organisms. We note that our predicted networks are 6–10x as dense as the signal-to-noise ratio (SNR) estimated in true PPI networks (1:1000, [93]); thus we still likely have false positive edges and spurious connections. Although our downstream clustering approach will help with denoising, or a higher threshold for interaction could be selected to more closely match this SNR, improvements in network inference will ultimately have the largest impact on downstream performance. Structure-based PPI methods such as AlphaFold-Multimer [27] remain too slow for most labs to apply at interactome scale, but any sufficiently fast PPI prediction method can be substituted into PHILHARMONIC, and should improve its performance as the accuracy of these fast PPI prediction methods advance. Moreover, it is well known that protein interaction networks differ across tissues [30], and that sub-cellular localization likewise plays a role in protein interaction [92]. Our current approach assumes a single PPI network, and orthogonal information such as localization prediction [81], gene co-expression, or tissue type could help further refine analysis of functional networks.

Non-model organisms play a crucial role in our world, from the impact of coral on reef ecosystems [66], to the value of livestock and crops in agriculture, to the potential of microbes to combat climate change through carbon capture [33] or digestion of plastics [23]. The broad applicability and ease of use of PHILHARMONIC will enable domain experts to organize the proteome into functional clusters, to identify testable functional hypotheses, and to dissect and understand the complex cellular systems of these organisms.

## Supplementary Material

Supplement 1
