# Supplementary material for: Decoding the Functional Interactome of Non-Model Organisms with PHILHARMONIC
Source: bioRxiv. 2025 Jan 14:2024.10.25.620267. Originally published 2024 Oct 29. Preprint. [Version 2] doi: 10.1101/2024.10.25.620267 (PMC11565725; doi:10.1101/2024.10.25.620267)
Supplement: Supplementary file 1 [file media-1.pdf]

A Appendix for Sledzieski et al. – PHILHARMONIC

A.1 Counts of PPI in different species

We constructed Figure A1 by downloading the specified versions of the BioGRID [70] database from `https://downloads.thebiogrid.org/BioGRID` and grouping interactions by species. We find substantial growth in the number of known human and yeast PPIs in the specified time frame, but relatively much less growth in even other model organisms, let alone non-model organisms.

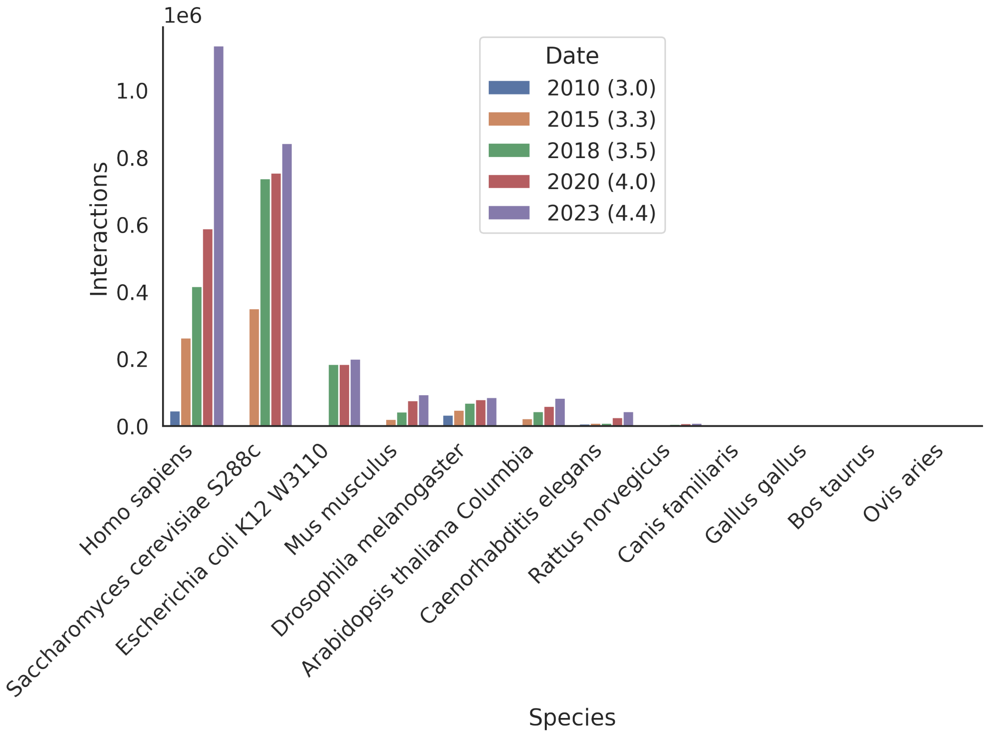

Figure A1: Known experimental PPIs are sparse outside of a small number of organisms. We show the number of experimental interactions in the BioGRID [70] database over the past $\approx$ 15 years, in several different organisms. While the number of known interactions has grown rapidly in humans and yeast, there remain very few known interactions in other species, and the number of known interactions is growing slowly.

A.2 The coral holobiont

Coral colonies are comprised of clonal cnidarian polyps that depend on a symbiotic relationship with algae in the family Symbiodiniaceae [53]. These dinoflagellate algae harvest light and synthesize nutrients in exchange for habitat and nitrogen sources [74]. Originally thought to primarily include endosymbiotic algae, the symbiosis is now known to extend to a much more complex community. The collective of thousands of bacteria, bacteriophages, viruses and fungi, in addition to Symbiodiniaceae and cnidarian, is known as the coral *holobiont* [93]. Mass coral bleaching, or the expulsion of the symbiotic algae due primarily to thermal stress driven by marine heatwaves, is resulting in substantial coral mortality [41]. A recent study assessed 100 worldwide locations and found that the annual risk of coral bleaching has increased from an expected 8% of locations in the early 1980s, to 31% in 2016 [41], and this was before some of the most acute heat waves of the early 2020s.

A.3 Implementation of PHILHARMONIC

We implement PHILHARMONIC using the Snakemake package. Figure A2 shows the entire pipeline, generated using the command `snakemake --configfile config.yml --filegraph | dot -Tpng > img/pipeline.png`.

This full figure shows not only the primary steps described in the main text, but additional necessary steps such as downloading required databases, preparing the hmm database, initial filtering and generating candidates for PPI prediction, and compiling all results together.

A.4 List of GO Slim terms for initial filtering

Table A1: GO Filter List. We select a subset of high-level GO Slim terms, and filter candidate proteins to any annotated with these terms and any of their children. This allows us to focus on a functionally interesting subset of the network. We allow the user to provide their own list of GO filters, or to not filter at all.

GO Term	Description
GO:0098754	detoxification
GO:0098542	defense response to other organism
GO:0050886	endocrine process
GO:0050877	nervous system process
GO:0048870	cell motility
GO:0042060	wound healing
GO:0023052	signaling
GO:0022600	digestive system process
GO:0015979	photosynthesis
GO:0012501	programmed cell death
GO:0007018	microtubule-based movement
GO:0006954	inflammatory response
GO:0006914	autophagy
GO:0006790	sulfur compound metabolic process
GO:0006766	vitamin metabolic process
GO:0006629	lipid metabolic process
GO:0006575	cellular modified amino acid metabolic process
GO:0006520	amino acid metabolic process
GO:0006486	protein glycosylation
GO:0006457	protein folding
GO:0006091	generation of precursor metabolites and energy
GO:0005975	carbohydrate metabolic process
GO:0003016	respiratory system process
GO:0003014	renal system process
GO:0003013	circulatory system process
GO:0003012	muscle system process
GO:0002376	immune system process
GO:1901135	carbohydrate derivative metabolic process
GO:0071941	nitrogen cycle metabolic process
GO:0061007	hepaticobiliary system process

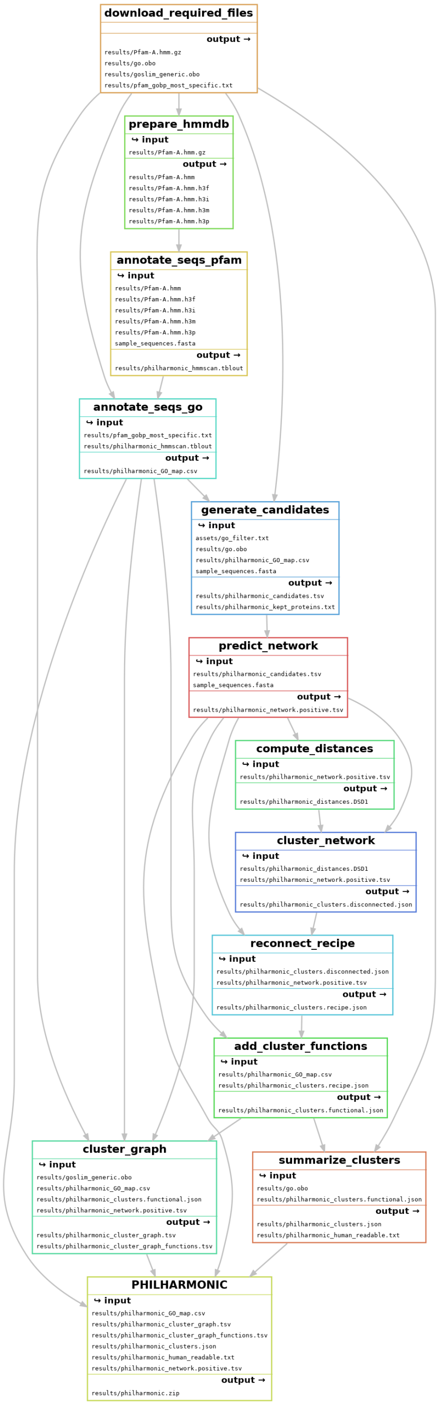

Figure A2: Full PHILHARMONIC implemented in Snakemake

A.5 Human-readable outputs

A.5.1 LLM Prompt

```python
task_instruction = """
You are an expert biologist with a deep understanding of the Gene Ontology. Your job is to give short, intuitive, high-level names to clusters of proteins, given a set of GO terms associated with the proteins and their frequencies.
"""

confidence_score = """
In addition to the name, you should indicate how confident you are that your label is correct, and that it is representative of the function of the cluster. This score should be None, Low, Medium, or High. If you cannot find a connection between the functions in the cluster, give a name of Unknown with a confidence of None.
"""

format_instruction = """
Your response should start with only the name of the cluster on the first line. Then, provide a short paragraph including your explanation for why you gave the cluster that name. Finally, on a new line, provide your confidence score. You should not put blank lines between these sections.
"""

analytical_approach = """
You should try to be as specific as possible in your naming, to avoid overlapping names with other similar clusters. However, the names should still be short and human readable, ideally fewer than 10 words. You should consider the most common GO terms in the cluster, and try to find a common theme or function that ties them together. If you cannot find a common theme, you should give the cluster a name of Unknown.
"""

one_shot_example = """
For example, given the cluster description below:

Cluster of 14 [pdam_00002129-RA,pdam_00001718-RA,...] (hash 1332063120138743063)
Triangles: 27.0
Max Degree: 8
Top Terms:
    GO:0071502 - <cellular response to temperature stimulus> (11)
    GO:0019233 - <sensory perception of pain> (11)
    GO:0042493 - <response to drug> (10)
    GO:0007603 - <phototransduction, visible light> (10)
    GO:0004876 - <complement component C3a receptor activity> (9)

We would name this cluster Temperature, Pain, and Drug Response because there is a high representation for GO terms related to temperature, drug, and pain response.
"""

request = """
Please name the following cluster:
"""

LLM_SYSTEM_TEMPLATE = (
    task_instruction
    + confidence_score
    + format_instruction
    + analytical_approach
    + one_shot_example
    + request
)
```

A.5.2 Sample PHILHARMONIC human-readable output.

All clusters are presented to the end user in a flat text file. The `hash` allows the cluster to be identified in the accompanying `.json` file. These summaries allow a user to easily scan through clusters and find communities they are interested in investigating further.

```
Cluster Name: Pain, Drug Response, and Development
Cluster of 20 proteins [pdam_00013683-RA, pdam_00006515-RA, pdam_00000216-RA, ...] (hash 208641124039621440)
20 proteins re-added by ReCIPE (degree, 0.75)
Edges: 3
Triangles: 0
Max Degree: 2
Top Terms:
        GO:0019233 - <sensory perception of pain> (20)
        GO:0048148 - <behavioral response to cocaine> (19)
        GO:0006468 - <protein phosphorylation> (19)
        GO:0007507 - <heart development> (19)
        GO:0010759 - <positive regulation of macrophage chemotaxis> (19)
        GO:0001963 - <synaptic transmission, dopaminergic> (19)
        GO:0071380 - <cellular response to prostaglandin E stimulus> (19)
        GO:0071502 - <cellular response to temperature stimulus> (19)
        GO:0008542 - <visual learning> (19)
        GO:0007601 - <visual perception> (19)
LLM Explanation: This cluster is characterized by a strong representation of GO terms associated with sensory perception of pain, responses to drugs (specifically cocaine), and developmental processes, especially in the heart. The presence of terms related to sensory response and nervous system functions, alongside those concerning cellular processes and behavioral responses, suggests a common role in both response to stimuli and the development of certain physiological traits.
LLM Confidence: High
```

A.5.3 Sample PHILHARMONIC JSON cluster.

The main output of PHILHARMONIC is a `.json` file containing a full specification of each cluster, including all members, proteins re-added by ReCIPE, the subgraph of edges in the cluster, GO term annotations, and all human-readable annotations.


```
...,
"208641124039621440": {
        "members": [
            "pdam_00013683-RA",
            "pdam_00006515-RA",
            "pdam_00000216-RA",
            "pdam_00009314-RA",
            "pdam_00024660-RA",
            "pdam_00021435-RA",
            "pdam_00000370-RA",
            "pdam_00023856-RA",
            "pdam_00022321-RA",
            "pdam_00016148-RA",
            "pdam_00006995-RA",
            "pdam_00019541-RA",
            "pdam_00014380-RA",
            "pdam_00000035-RA",
            "pdam_00003202-RA",
            "pdam_00007455-RA",
            "pdam_00017375-RA",
            "pdam_00006721-RA",
            "pdam_00003531-RA",
            "pdam_00022374-RA"
        ],
        "graph": [
            [
```

```
                "pdam_00022374-RA",
                "pdam_00023856-RA",
                0.5334205031394958
            ],
            [
                "pdam_00022374-RA",
                "pdam_00000035-RA",
                0.6888012290000916
            ],
            [
                "pdam_00007455-RA",
                "pdam_00023856-RA",
                0.6809149384498596
            ]
        ],
        "recipe": {
            "degree": {
                "0.75": [
                    "pdam_00021087-RA",
                    "pdam_00012633-RA",
                    "pdam_00011773-RA",
                    "pdam_00012527-RA",
                    "pdam_00003878-RA",
                    "pdam_00018049-RA",
                    "pdam_00018748-RA",
                    "pdam_00002992-RA",
                    "pdam_00021189-RA",
                    "pdam_00017594-RA",
                    "pdam_00013619-RA",
                    "pdam_00008678-RA",
                    "pdam_00006058-RA",
                    "pdam_00002321-RA",
                    "pdam_00015309-RA",
                    "pdam_00020434-RA",
                    "pdam_00008656-RA",
                    "pdam_00014328-RA",
                    "pdam_00011829-RA",
                    "pdam_00006524-RA"
                ]
            }
        },
        "GO_terms": {
            "GO:0030168": 18,
            "GO:0002032": 18,
            "GO:0022400": 18,
            .
            .
            .
            "GO:0008345": 2,
            "GO:0035269": 1,
            "GO:0046329": 1,
            "GO:0060049": 1
        },
        "human_readable": "Cluster Name: Cell Signaling and Regulation\nCluster of 20 proteins [
    pdam_00021685-RA, pdam_00003645-RA, pdam_00012637-RA, ...] (hash 2185119890364449780)\n20
    proteins re-added by ReCIPE (degree, 0.75)\nEdges: 6\nTriangles: 0\nMax Degree: 4\nTop Terms
    :\n\t\tGO:0030168 - <platelet activation> (19)\n\t\tGO:0002032 - <obsolete desensitization of
     G protein-coupled receptor signaling pathway by arrestin> (19)\n\t\tGO:0022400 - <regulation
     of opsin-mediated signaling pathway> (19)\n\t\tGO:0051586 - <positive regulation of dopamine
     uptake involved in synaptic transmission> (19)\n\t\tGO:0031635 - <adenylate cyclase-
    inhibiting opioid receptor signaling pathway> (19)\n\t\tGO:2000479 - <regulation of cAMP-
    dependent protein kinase activity> (19)\n\t\tGO:0040015 - <negative regulation of
    multicellular organism growth> (19)\n\t\tGO:0072224 - <metanephric glomerulus development>
    (19)\n\t\tGO:0070963 - <positive regulation of neutrophil mediated killing of gram-negative
    bacterium> (19)\n\t\tGO:0035025 - <positive regulation of Rho protein signal transduction>
```

```
    (19)\nLLM Explanation: This cluster consists of proteins that are highly associated with
    various signaling pathways and regulatory processes, particularly related to cell activation
    and communication. The predominance of GO terms related to platelet activation, GPCR
    signaling, dopamine uptake regulation, and various regulatory mechanisms suggests a strong
    focus on how cells interact and respond to stimuli, which includes growth regulation and
    immune function as well.\nLLM Confidence: High\n",
        "llm_name": "Cell Signaling and Regulation",
        "llm_explanation": "This cluster consists of proteins that are highly associated with
    various signaling pathways and regulatory processes, particularly related to cell activation
    and communication. The predominance of GO terms related to platelet activation, GPCR
    signaling, dopamine uptake regulation, and various regulatory mechanisms suggests a strong
    focus on how cells interact and respond to stimuli, which includes growth regulation and
    immune function as well.",
        "llm_confidence": "High"
    }...
```

A.6 Supplemental material for the *P. damicornis* network

We show detailed statistics of the *P. damicornis* network, including numbers of nodes, edges, median and average degrees, and sparsity in Table A2.

Table A2: PHILHARMONIC predicted network statistics. We run PHILHARMONIC on three species– the coral *P. damicornis*, its symbiont *C. goreaui*, and the fruit fly *D. melanogaster*. Here, we show basic statistics of the predicted network for each species.

	P. damicornis	*C. goreaui*	*D. melanogaster*
**Proteins**	7,267	8,204	4,192
**Edges**	348,278	568,536	197,510
**Med. Degree**	37	44.5	45.5
**Avg. Degree**	95.852	138.560	94.232
**Sparsity**	0.00656	0.00845	0.01124

A.6.1 Functional Coherence Analysis

In Figure 2, we compute the Jaccard similarity between pairs of proteins over the full set of GO terms. The Jaccard similarity is defined as

$$J(A, B) = \frac{A \cap B}{A \cup B} \tag{3}$$

where A, B are the sets of GO terms assigned to each individual protein. In Figure A3, we use only GO Slim terms to compute similarity between proteins. We find that even over that reduced set, PHILHARMONIC clusters are still significantly more functionally coherent than would be expected at random.

A.6.2 Comparison with other clustering methods

In Section A.8.1, we show that DSD + hierarchical spectral clustering + ReCIPE results in clusters that are significantly functionally coherent. Here, we compare with several other state-of-the-art community detection methods, evaluating them by the same metric—the extent to which clusters detected by the given algorithm share more function that would be expected by random clustering.

Using the CDlib Python package (version 0.4.0) [76], we evaluate several different algorithms for generating both overlapping and non-overlapping clusters. In Table A3, we show both the number of clusters detected by this method, and the p-value of the one-tailed T-test evaluating cluster coherence of computed vs. random clusters. Only regularized spectral clustering [106] and IPCA [57] have performance competitive with ours. In Figure A4, we show the distributions of cluster sizes and cluster coherences for each method. Only our method produces clusters with uniform size at a size which is easily amenable to biological discovery—both regularized spectral and IPCA result in many very large or small clusters that are difficult to interpret.

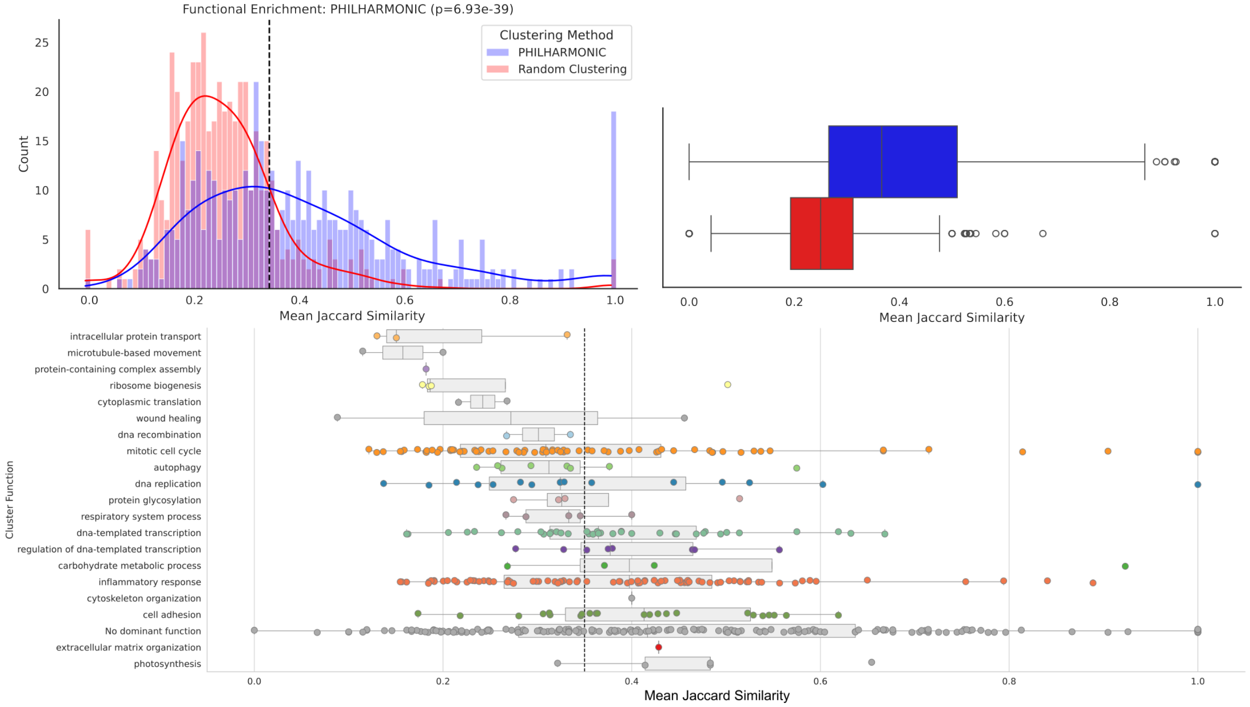

Figure A3: *P. damicornis* functional coherence analysis using limited set of GO Slim terms. We perform the same analysis as in Figure 2a,b using only the subset of GO terms from GO Slim. We find that PHILHARMONIC clusters are still significantly functionally enriched, although with a higher threshold to clearly separate non-random cluster coherences.

A.6.3 Singular value dominance of gene expression within clusters

In Figure 2d, we compute the coherence of a cluster by the pairwise co-expression of proteins within the cluster. Here, rather than looking at the average of several pairwise correlations, we instead subset the gene expression to include only the genes corresponding to proteins within a given cluster, and compute the singular values of this matrix. Then, we look at the dominance of the first singular value—where a high first singular value corresponds with strongly coordinated expression within the cluster. In Figure A5, we show that PHILHARMONIC clusters likewise have significantly more coordinated expression than random clusters.

A.6.4 Thermal response cluster

The majority the proteins in the cluster are predicted to be GPCRs, which are predicted to bind to various subsets and likely modulate the functions of the diverse ion channels described in Section 3.4.1, in response to different stimuli. These proteins include `pdam_00021189-RA`, with a sequence signature somewhat similar to the alpha-2Da adrenergic receptor detected in the zebrafish brain [78], and `pdam_00001720-RA`, predicted by sequence homology to be an allatostatin-A receptor. The neuropeptide allatostatin-A (AstA) and its cognate receptors (AstARs) are involved in the modulation of feeding behavior in mosquitos. Two of the other proteins `pdam_00006261-RA` (most likely an orexin receptor) and `pdam_00013140-RA` (neuropeptide FF receptor 2-like) are both similar to receptor proteins that regulate the feeding process in fish [58]. `pdam_00023837-RA`, `pdam_00016481-RA` and `pdam_00016115-RA` round out the collection of GPCRs. Finally, there are three proteins in the cluster which are not either ion channels or GPCRs: `pdam_00019706-RA`, predicted to be a fibroblast growth factor, `pdam_00004445-RA`, predicted to be a zinc transporter, and `pdam_00017094-RA` a completely uncharacterized protein with no homology to any proteins of known function.

Below, we show a full list of cluster membership and their best homology match from a BLAST search. We also show ColabFold [64] alignments, pLDDT plots, and PAE plots for each predicted structure.

- pdam_00001720-RA: allatostatin-A receptor-like

Table A3: Use of other community detection methods for network clustering. Only regularized spectral clustering (similar to a subroutine of our approach) and IPCA approach clustering results competitive with PHILHARMONIC. We show both methods for strictly non-overlapping clusters (first section) and methods which allow overlap (second section).

Clustering Method	Number of Clusters	p-value
Ours (DSD + Hierarchical Spectral + ReCIPE)	468	2.668e-48
Eigenvector [67]	6	1.420e-01
Infomap [77]	53	9.908e-07
Label propagation [21]	27	1.590e-03
Leiden [96]	8	6.022e-02
Louvain [11]	9	6.342e-02
Regularized spectral [106]	440	7.578e-41
Congo [33]	*Out of memory*	*>350 GB*
IPCA [57]	2312	0.0
LFM [54]	*Time out*	*>24 hours*
Multicom [39]	6	2.990e-02
Walkscan [38]	1	-

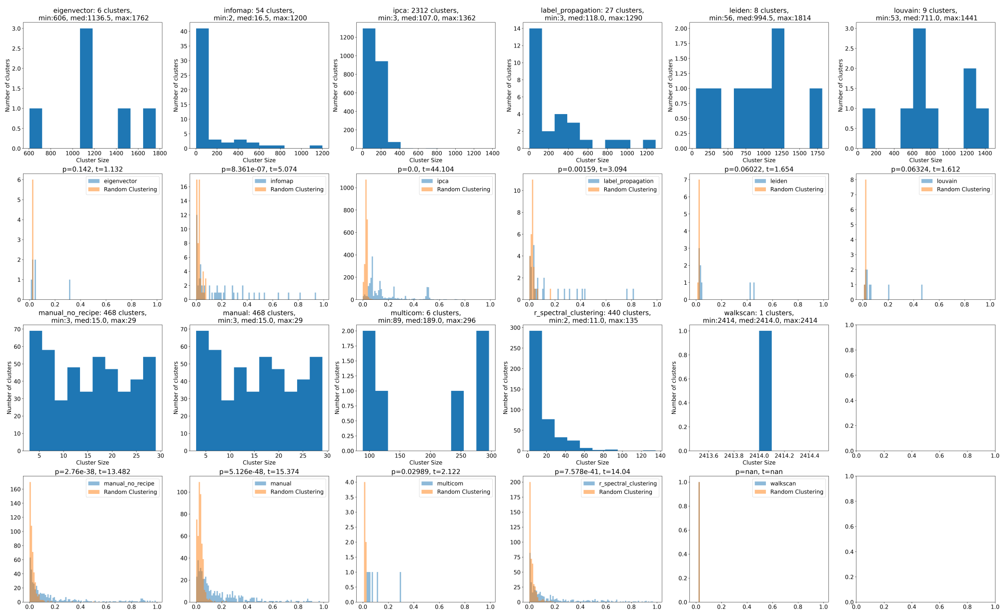

Figure A4: Comparison of cluster sizes with other community detection methods. While IPCA and spectral clustering have competitive functional coherences, the distribution of cluster sizes makes it much more difficult to investigate the biological relevance of a given cluster.

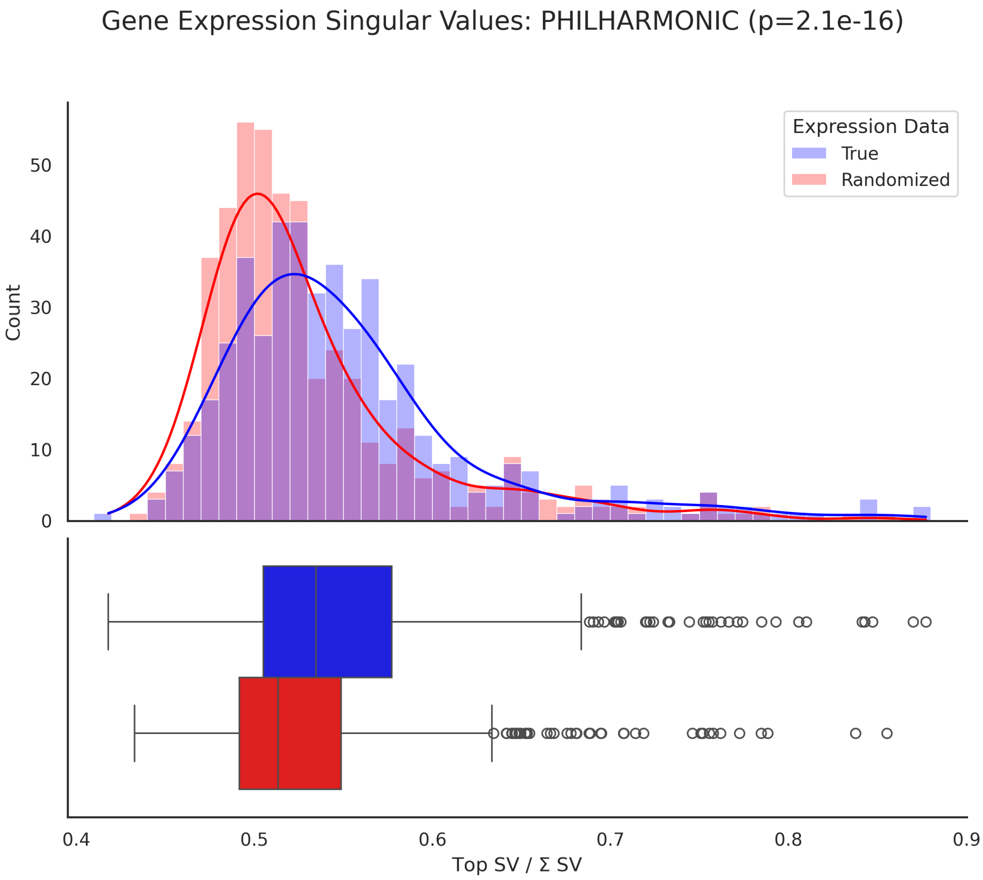

Figure A5: Cluster coherence by singular value of gene expression Similar to Figure 2, but with co-expression calculated using the dominance of the first singular value.

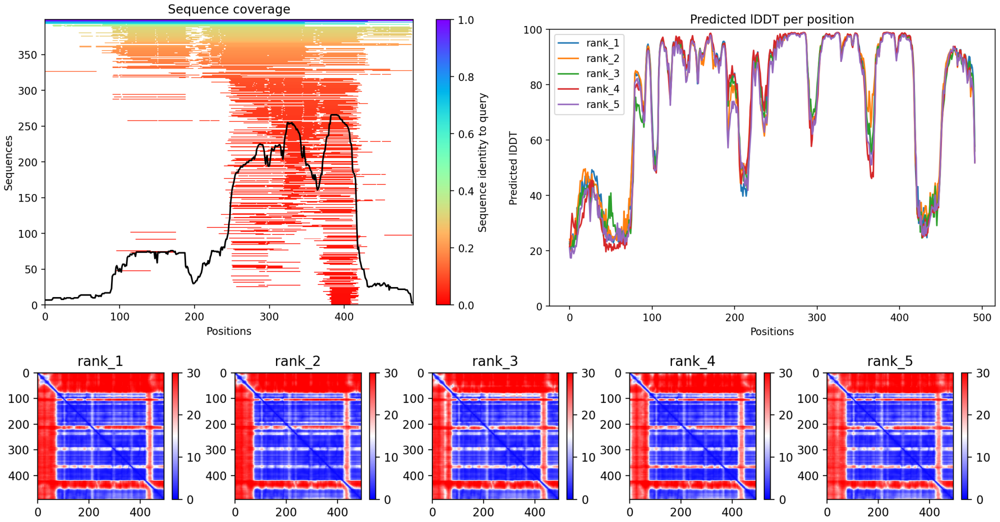

Figure A6: ColabFold alignments, pLDDT, and PAE for pdam_00017094

- pdam_00004445-RA: zinc transporter 8-like
- pdam_00005806-RA: potassium/sodium hyperpolarization-activated cyclic nucleotide-gated channel 2-like
- pdam_00006261-RA: orexin receptor type 1-like
- pdam_00008678-RA: cyclic nucleotide-gated channel rod photoreceptor subunit alpha-like
- pdam_00010576-RA: TWiK family of potassium channels protein 7-like isoform X1
- pdam_00013140-RA: neuropeptide FF receptor 2-like
- pdam_00016115-RA: beta-2 adrenergic receptor-like
- pdam_00016481-RA: beta-1 adrenergic receptor-like
- pdam_00017094-RA: **Uncharacterized**
- pdam_00019465-RA: potassium voltage-gated channel subfamily A member 7-like
- pdam_00019542-RA: potassium voltage-gated channel protein Shal-like isoform X1
- pdam_00019706-RA: fibroblast growth factor 2-like isoform X2
- pdam_00021189-RA: alpha-2Da adrenergic receptor-like
- pdam_00023837-RA: histamine H2 receptor-like, partial

A.6.5 Environmental stimuli cluster

Below, we show a full list of cluster membership and their best homology match from a BLAST search.

- pdam_00001381-RA: potassium voltage-gated channel subfamily D member 3-like
- pdam_00001388-RA: gamma-aminobutyric acid receptor alpha-like
- pdam_00001389-RA: gamma-aminobutyric acid receptor subunit alpha-2-like

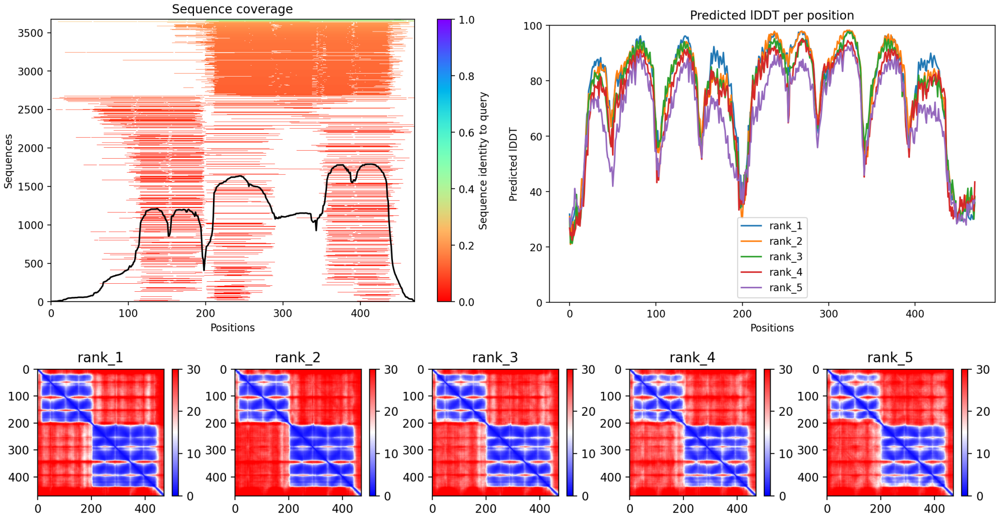

Figure A7: ColabFold alignments, pLDDT, and PAE for pdam_00001720

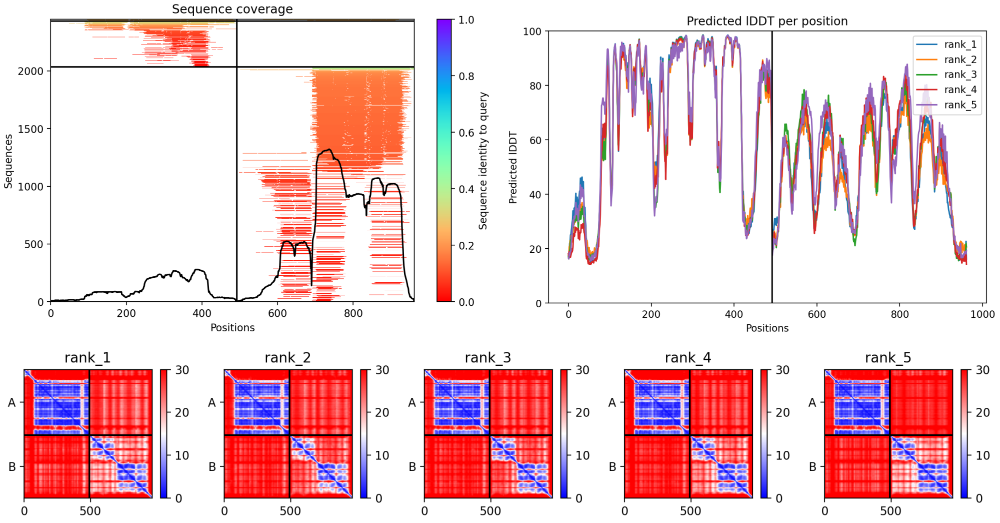

Figure A8: ColabFold alignments, pLDDT, and PAE for pdam_00017094 and pdam_00001720 in complex

- pdam_00001924-RA: glycine receptor subunit alphaZ1-like
- pdam_00003290-RA: transient receptor potential cation channel subfamily V member 6-like
- pdam_00003714-RA: histone deacetylase 1-like
- pdam_00006109-RA: **Uncharacterized**
- pdam_00006156-RA: calcium-independent phospholipase A2-gamma-like
- pdam_00006320-RA: potassium voltage-gated channel subfamily A member 2-like
- pdam_00006972-RA: neuronal acetylcholine receptor subunit alpha-10-like
- pdam_00006973-RA: neuronal acetylcholine receptor subunit alpha-10-like isoform X1
- pdam_00008620-RA: FGFR1 oncogene partner 2 homolog
- pdam_00011550-RA: **Uncharacterized**
- pdam_00011780-RA: lipid droplet-associated hydrolase-like
- pdam_00012376-RA: gamma-aminobutyric acid receptor subunit beta-2-like
- pdam_00012507-RA: neuronal acetylcholine receptor subunit alpha-5-like
- pdam_00015999-RA: neuronal acetylcholine receptor subunit alpha-7-like isoform X2
- pdam_00018139-RA: **Uncharacterized**
- pdam_00021982-RA: phosphatidylinositol phosphatase PTPRQ-like
- pdam_00024051-RA: gamma-aminobutyric acid receptor alpha-like

A.6.6 g:Profiler analysis of clusters

We map `pdam` protein identifiers to ENSEMBL gene identifiers using the ENSEMBL *P. damicornis* reference genome, downloaded from `http://ftp.ensemblgenomes.org/pub/metazoa/release-59/fasta/pocillopora_damicornis_gca003704095v1/pep/Pocillopora_damicornis_gca003704095v1.ASM370409v1.pep.all.fa`. This file contains both protein keys (`XP_*`) and gene (`LOC*`) identifiers. We create a BLAST database from these sequence query our set of *P. damicornis* proteins against this database. This allows to map our IDs to the `LOC*` identifiers needed to query g:Profiler [50]. We use version 1.0.0 of the `gprofiler-official` Python package.

A.6.7 Structural analysis of uncharacterized proteins

We predict 3D structures for several proteins of interest using ColabFold version 1.5.5 [64], run with default parameters. For single sequences, `alphafold2_ptm` was used, while `alphafold2_multimer_v3` was used for pairs of sequences.

A.7 Dissecting the *C. goreaui* network

We show the symbiont cluster graph in Figure A9, along with node degree and cluster size distributions. We show detailed statistics of the *C. goreaui* network in Table A2. Below, we replicate the functional coherence analysis (Section A.7.1) and highlight two clusters related to cellular oxidant de-toxification (Section A.7.2).

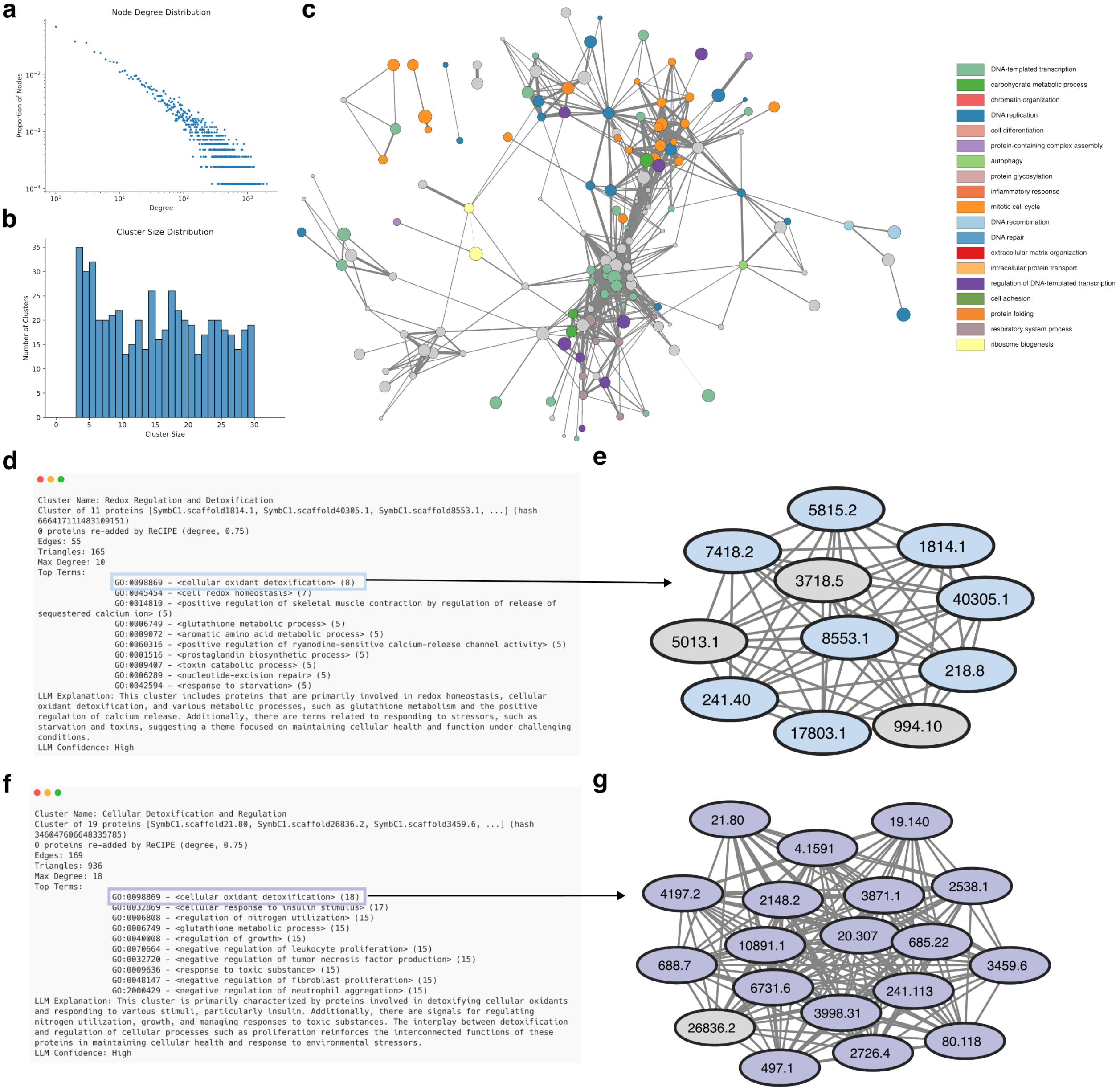

Figure A9: Dissecting the functional network of the symbiont *C. goreaui*. **(a)** Node degree distribution of predicted network. **(b)** Size distribution of PHILHARMONIC clusters. **(c)** Cluster graph, shown with $t = 50$ connecting edges. **(d)** Human-readable cluster description for the cluster of 11 described in Section A.7.2. **(e)** Graph for this cluster, colored by annotation for cellular oxidant detoxification (GO:0098869). **(f)** Human-readable cluster description for the cluster of 19 described in Section A.7.2. **(e)** Graph for this cluster, colored by annotation for cellular oxidant detoxification (GO:0098869).

A.7.1 Functional coherence in *C. goreaui*

We perform the same analysis as in Section 3.1 on the symbiont *C. goreaui* to check whether functional coherence is preserved. We likewise find significant functional coherence in PHILHARMONIC clusters ($p = 2.74 \times 10^{-44}$). We show the results of this analysis using all GO terms in Figure A10; we find similar results using GO Slim terms.

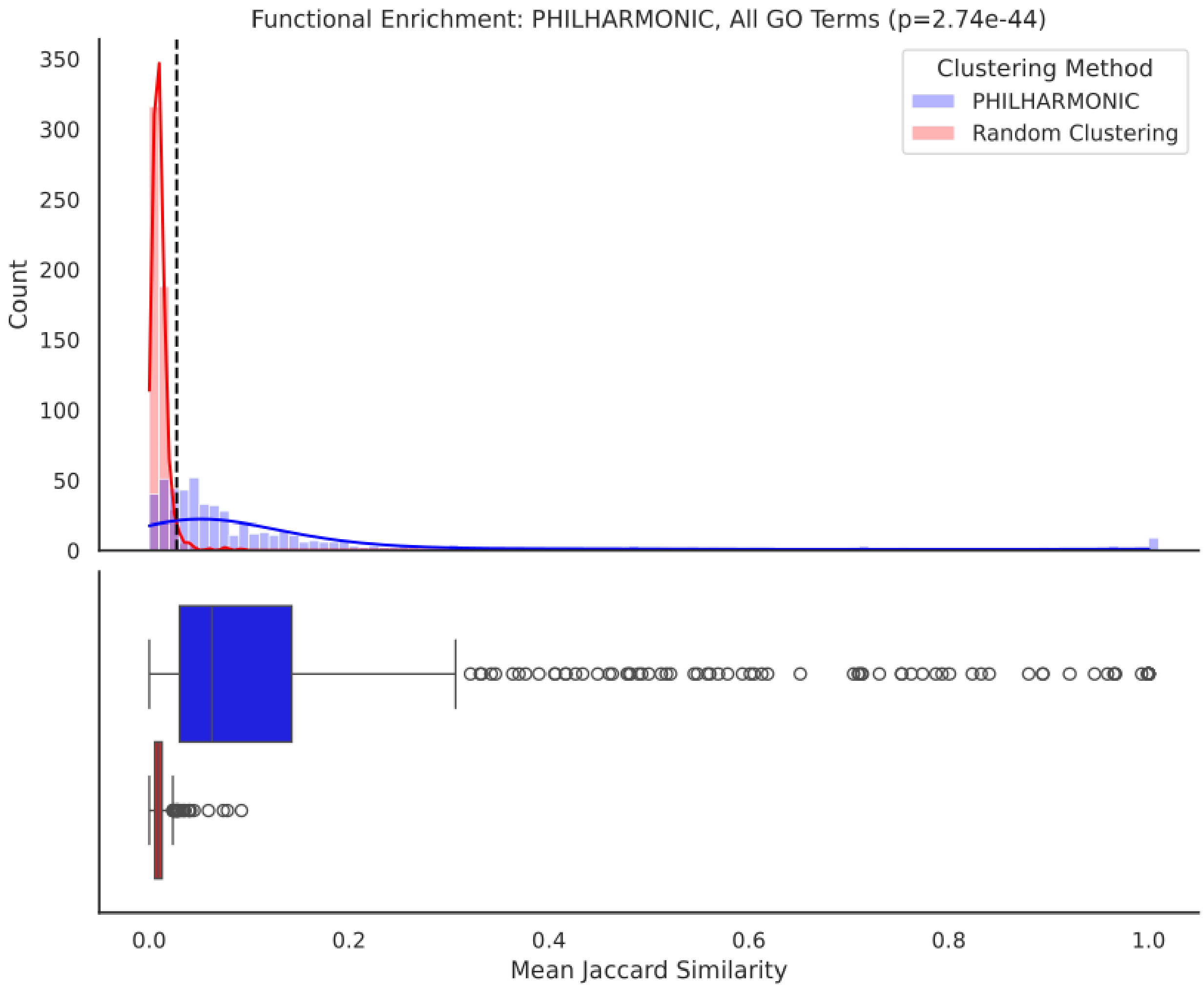

Figure A10: Functional coherence analysis using all GO terms in *C. goreaui*.

A.7.2 Oxidative Stress in *C. goreaui*

These two clusters (Cluster of 11 and Cluster of 19) contain many putative stress response genes. In Figure A9 we show the PHILHARMONIC human-readable output (A9d,A9f) and cluster graphs (A9e,A9g). The cluster of 11 (A9e, blue) includes 3 probable glutathione S-transferase (GST) proteins (1814.1, 218.8, 7418.2) as well as 5 likely glutaredoxin proteins (40305.1, 8553.1, 241.40, 5815.2, 17803.1). The cluster of 19 (A9g, purple) includes 7 probable GST proteins (3459.6,6731.6,20.307, 2148.2,10891.1,4197.2, 497.1), and 3 possible additional GST proteins (2726.4, 80.118, 19.140), as well as 2 probable glutaredoxin proteins (3871.1,685.22). GST and glutaredoxin proteins are known to be part of antioxidant response pathways to oxidative stress [10, 35], with glutaredoxins hypothesized to play a particularly important role in response to photo-oxidative stress in photosynthesizing plants [79]. In addition to the GST and glutaredoxim proteins, the cluster of 11 also contains three cytochrome proteins (3718.5, 5013.1, 994.10),one of which is a likely cytochrome-450 protein, where this family of proteins is generally preserved from algae to higher plants and can oxidize endogenous substrates in various biosynthetic pathways as well as xenobiotic substrates, in particular herbicides [71]. Other proteins in the cluster of 19 (purple) include proteins with remote homology to the YghU protein (3998.31,4.1591,688.7), one similar to the related yfcG protein, and a methionine sulfide reductase protein (21.80) These proteins all have been identified as modulating the response to oxidative stress, where YghU and yfcG appear to be a novel form of GST proteins [46], and methionine sulfide reductase proteins repair oxidized proteins and are protective against damage caused by oxidative stress [56]. We hypothesize that these two clusters are important for reaction to oxidative stress in the symbiont.

A.8 Dissecting the *D. melanogaster* network

We show the node degree distribution, cluster size distribution, and cluster graph for the *D. melanogaster* PHILHARMONIC network and clusters in Figure A11. We show detailed statistics of the fruit fly network in Table A2. We show that PHILHARMONIC clusters are still significantly coherent when using ground truth GO function annotations from FlyBase [94], rather than the GO terms transferred by PHILHARMONIC in Secion A.8.1. Below, we perform a case study of a cluster in fly that demonstrates the additional value that PHILHARMONIC brings in addition to node-level functional annotation (Section A.8.4).

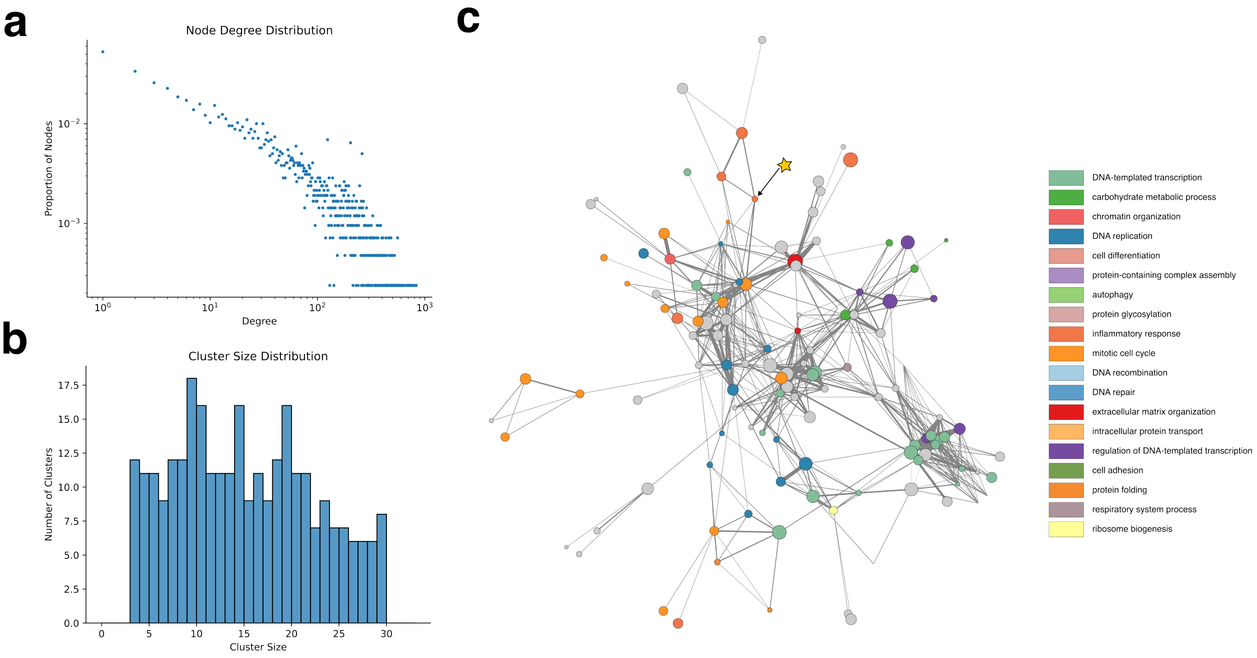

Figure A11: Dissecting the functional network of the fruit fly *D. melanogaster*. **(a)** Node degree distribution of predicted network. **(b)** Size distribution of PHILHARMONIC clusters. **(c)** Cluster graph, shown with $t = 50$ connecting edges.

A.8.1 Functional coherence in the fly

We perform the same analysis as in Section 3.1 and Section A10 on the fruit fly *D. melanogaster* network, with one important difference. While we were previously using GO function labels assigned by PHILHARMONIC (hmmscan + GODomainMiner), as an external validation we here use GO function annotations from FlyBase [94]. Using these gold-standard functional annotations, we likewise find significant functional coherence in PHILHARMONIC clusters ($p = 5.2 \times 10^{-16}$) in the fruit fly. We show the results of this analysis using all GO terms in Figure A12; we find similar results using GO Slim terms.

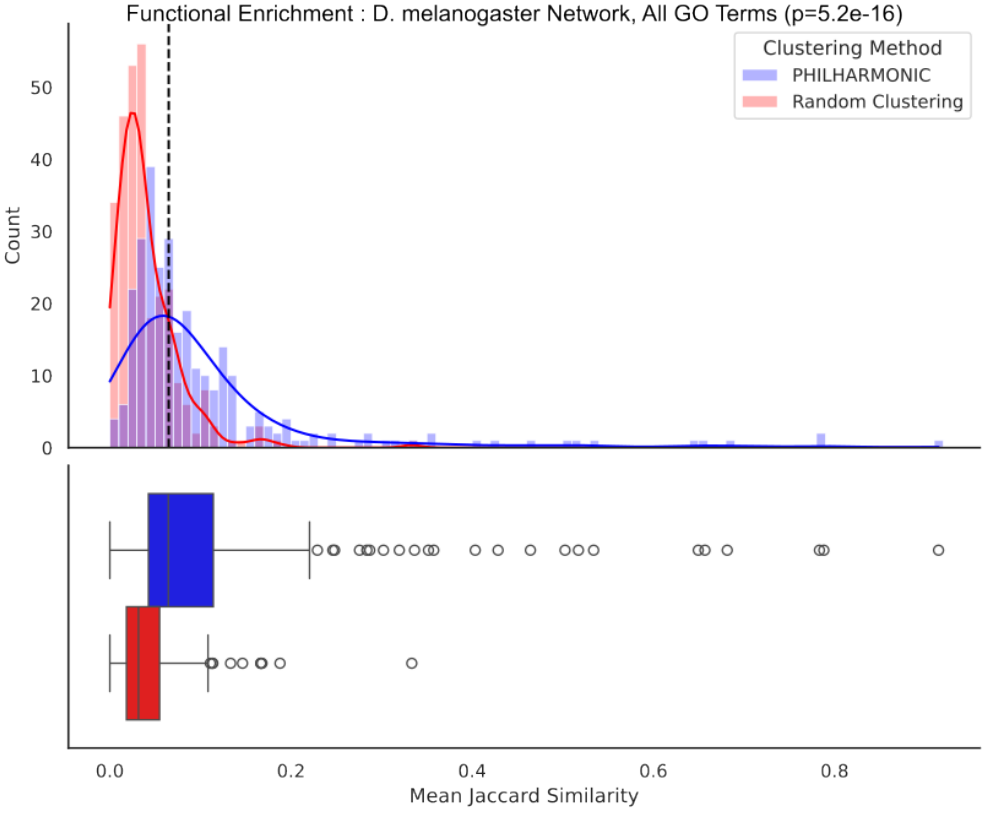

Figure A12: PHILHARMONIC discovers highly functional clusters in *D. melanogaster.* PHILHARMONIC clusters are significantly more functionally coherent compared to random clusters ($p = 5.2 \times 10^{-16}$, one-tailed independent t-test). Here, we are using ground truth GO functional annotations from FlyBase [94].

A.8.2 Effect of network noise on cluster functional coherence

While D-SCRIPT has been shown to achieve high accuracy in cross-species PPI prediction [85], it is likely that the predicted network contains noisy or missing edges. To isolate the effects of our downstream framework from the potential of noise in predicted edges, we substitute the network prediction step of PHILHARMONIC with the gold-standard *D. melanogaster* PPI network from STRING [89]. In Figure A.8.2, we show the results of our clustring and functional coherence analysis on this network. As expected, clusters display stronger coherence when the underlying network is less noisy, underscoring the need for continued improvement of PPI prediction methods and the potential for additional performance gains of the PHILHARMONIC method.

A.8.3 Functional coherence by shared FlyBase pathways

In Section A.8.1, we compute cluster coherence using true GO terms from FlyBase [94]. Here, we investigate a higher level of function, specifically that of shared pathways. FlyBase reports gene groups such as ”Proton-Coupled Amino

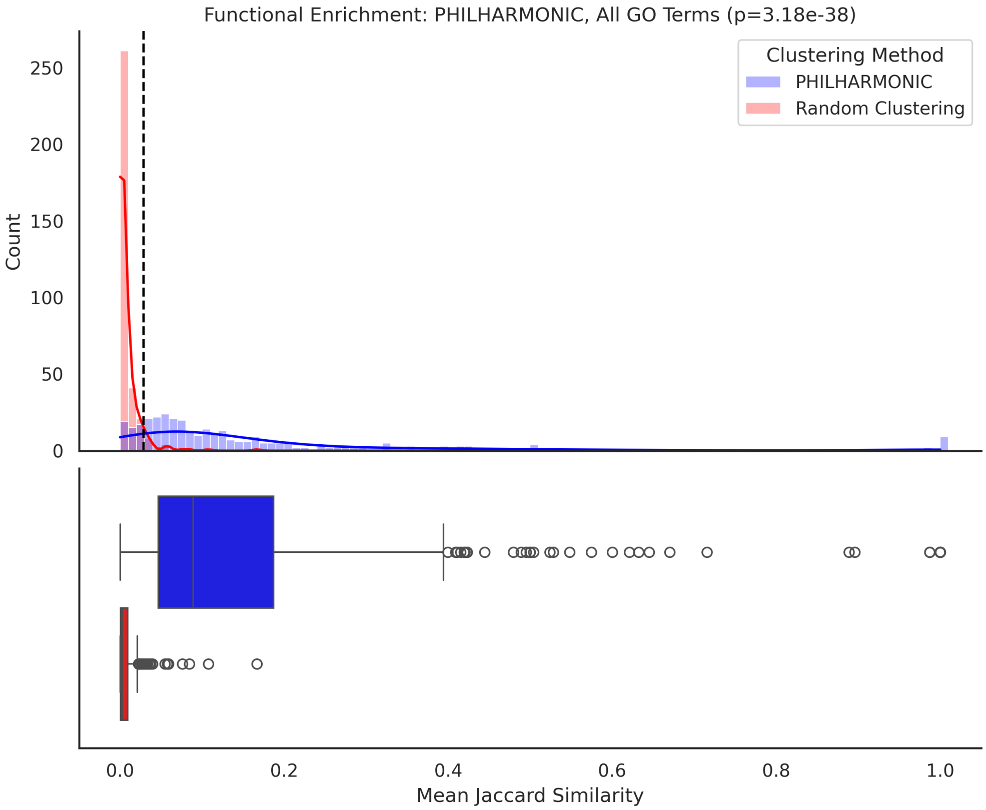

Figure A13: PHILHARMONIC performs better with a higher-confidence network. Compared to the results in Figure A12, PHILHARMONIC clusters are significantly more coherent when the true STRING PPI network is used, rather than a predicted network. We emphasize that this is not a possibility for many species, where a high-confidence PPI network is not available.

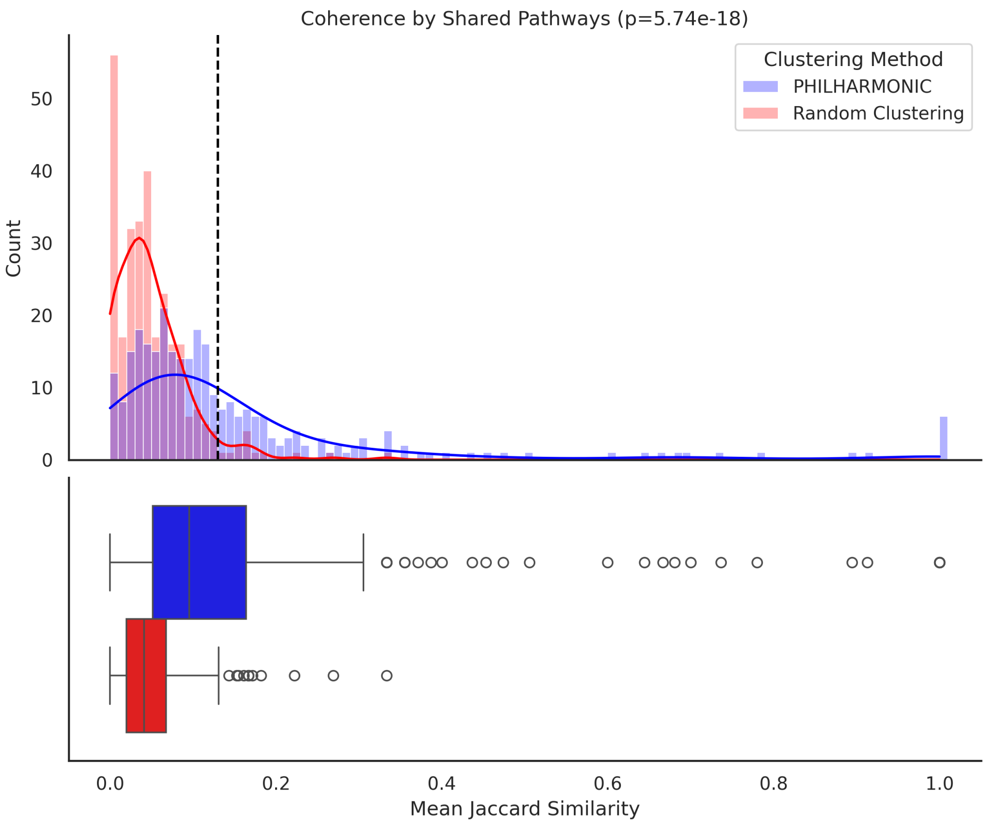

Figure A14: Jaccard similarity of shared FlyBase pathways. Cluster coherence is here computed not by shared GO terms, but by shared annotation of FlyBase gene groups.

Acid Transporters" or "Paired Homeobox Transcription Factors". We perform a similar analysis wherein we identify 853 gene groups annotated for at least one protein in our network. Then, we compute similarity within a cluster by the Jaccard similarity between sets of flybase gene groups, referred to here as "shared pathways." We show in Figure A14 that PHILHARMONIC clusters are significantly more likely to contain pairs of genes with shared pathways than random.

A.8.4 Case study: harnessing the cluster graph to understand higher-level functional organization

We demonstrate in the well-studied fly network that the high-level cluster graph can yield insights that cannot be obtained by looking only at individual proteins or clusters. We focus on a cluster of 9 proteins in the *D. melanogaster* network (Figure A15 inset). This cluster was specifically selected based on the high-level organization seen in the cluster graph (Figure A11, starred), as it is highly central in the graph and connects a diverse set of functions—what we dub a "bridge" cluster. We confirm this by computing the betweenness centrality for all nodes in the graph, where the selected cluster ranks 16th out of 285 clusters. In Figure A15, we show all nodes that have at least 10 crossing edges to the selected cluster. Proteins in this cluster interact with proteins annotated for inflammatory response (dark orange, GO:0006954), mitotic cell cycle (orange, GO:0000278), extracellular matrix organization (red, GO:0030198), DNA replication (dark blue, GO:0006260), carbohydrate metabolic process (green, GO:0005975), and DNA-templated transcription (pale green, GO:0006352) among others. In addition, this cluster is connected to several grey clusters, which themselves contain proteins with diverse functional annotations.

Below, we show a full list of cluster membership, both the gene name and the FlyBase identifier. This cluster is primarily composed of Gα subunits and the Gα-like *concertina*/Gα11/12. Looking at the literature, we find that Gαi and Gαo proteins are known to be involved in hearing [6] and give differential response in response to food deprivation [80]. The Gαs protein is involved in olfactory signal transduction [26] and Gαq is known to play a role in

photoreceptor signaling [34, 51] and the gustatory response [66].

Interestingly, this cluster also contains the DJ genes, that have been shown to convey a neuroprotective role against oxidative stress in multiple contexts [15, 103]. There is some evidence that Gαi and Gαo are target proteins of reactive oxygen species [69] making this cluster a good starting place to begin to look for mechanism. In general, we hypothesize that the proteins in this cluster are taking in environmental signals and routing them to the appropriate systems to respond, acting as a sort of "conductor" of cellular function.

- 7227.FBpp0070550: AstA-R1
- 7227.FBpp0072053: Gαs
- 7227.FBpp0076643: Gαi
- 7227.FBpp0085065: DJ-1β
- 7227.FBpp0086741: DJ-1α
- 7227.FBpp0087361: Gαo
- 7227.FBpp0110121: Gαq
- 7227.FBpp0291583: Gαq
- 7227.FBpp0300610: Concertina

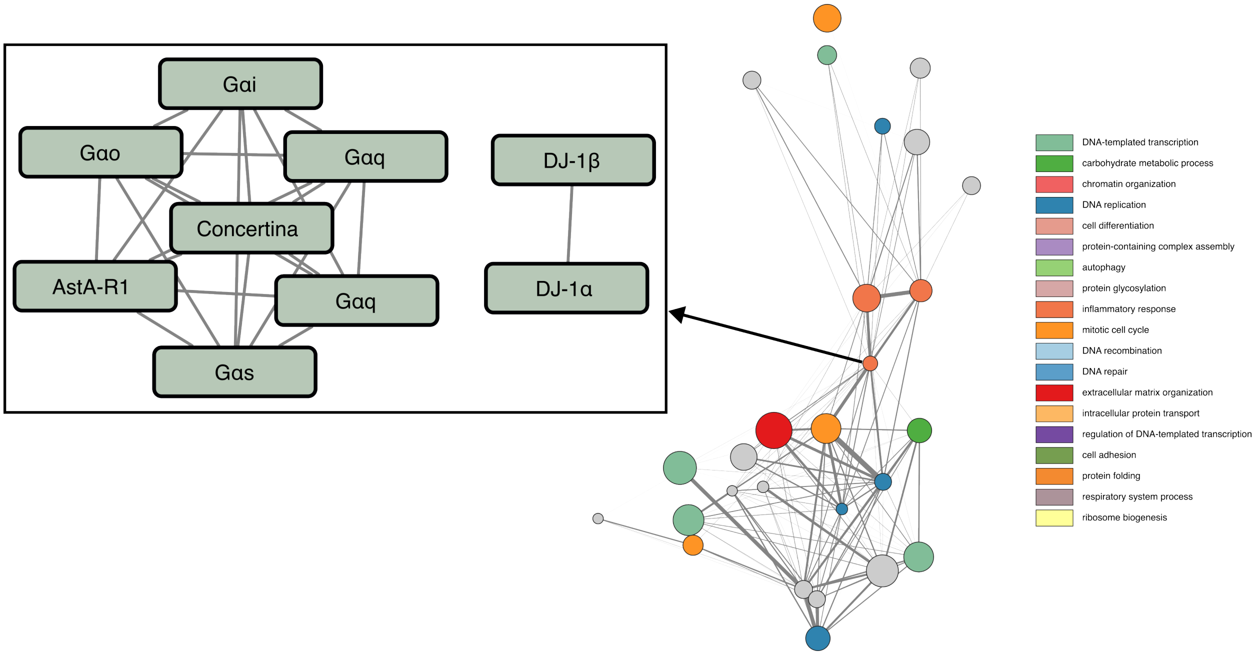

Figure A15: Signal routing in *D. melanogaster*. Here we show clusters that are connected to a central "bridge" cluster, shown in detail in the cutout. This cluster contains several G protein alpha subunits, as well as AstA-R1 and DJ-1α, and is connected to a diverse set of functional clusters. This cluster has one of the highest betweenness centralities in the cluster graph (Rank 16 out of 285 clusters; Betweeness centrality score: 0.0291)

A.9 PHILHARMONIC is robust to variation in hyper-parameters

We perform an in-depth evaluation of the quality of PHILHARMONIC clusters with a wide variety of different parameter settings both for clustering and for re-connection with ReCIPE. Specifically, we evaluate combinations of the following parameters:

- Initial number of clusters: [50, 100, 500]
- Cluster divisor (at each iteration): [5, 10, 20]
- Minimum cluster size: [3, 10]
- ReCIPE linear ratio: [0.1, 0.25]
- ReCIPE cthresh: [0.25, 0.5, 0.75]
- ReCIPE maximum proteins re-added: [10, 20, 50]

We provide the full set results in the attached Supplementary Sheet S1. Broadly, we find that PHILHARMONIC yields functionally coherent clusters across many parameter settings. We do identify a failure mode for PHILHARMONIC—specifically, if the cluster division at each step is too small (5), while at the same time the minimum cluster size is too large (10), we end up creating many small clusters in the end, which are then filtered out resulting in no clusters being returned. Provided that a low enough minimum size is set (we recommend 3) or a large split at each step (≥ 10), this outcome can be avoided. In future work, we will explore methods for automatically detecting and correcting this failure mode.

A.10 Pseudocode for the ReCIPE Algorithm

```
def reconnect(clusters, linear_ratio=0.1, cthresh=0.75, max_proteins=20, metric="min degree"):
    all_added_proteins = {}
    for cluster in clusters:
        added_proteins = {}
        potential:= proteins not in cluster
        sort potential proteins by metric

        num_proteins:= proteins in cluster

        num_components:= connected components in cluster
        percent_connectivity = 1 - (num_components - 1) / (num_proteins - 1)

        while (len(potential) > 0) and (percent_connectivity < cthresh):
            connection_minimum = linear_ratio * num_components

            for prot in potential:
                prot_degree:= number of components prot connects

                if prot_degree >= connection_minimum
                    add prot to added_proteins
                    recompute num_components
                    recompute percent_connectivity
                    remove prot from potential

                if len(added_proteins) > max_proteins:
                    break

        add (cluster, added_proteins) to all_added_proteins

    return all_added_proteins
```

A.11 ReCIPE DREAM Networks Analysis

We use three networks from the DREAM challenge to validate ReCIPE performance. DREAM 1 is a PPI network representing protein interactions and functional association, which is derived from the STRING database [90]; the network has 17,388 proteins and 1,973,788 edges. DREAM 2 is a PPI network that only reflects physical protein networks, which is derived from the InWeb database [59]; the network has 12,325 proteins and 397,254 edges. DREAM 3

is a signaling network, which is curated from 27 sources from OmniPath [97], and contains 5,009 proteins and 18,270 edges. We show statistics for all three of these networks in Table A4.

In Figure A16, we show the function prediction results for all three networks computed by two methods. For both methods, we use the FUNC-E [30] package to compute a set of enriched GO terms for a cluster. We assign a cluster all enriched terms, and assign those functions to all held out proteins in that cluster. We then compute the Jaccard similarity (Figure A16a,b,c) or F1 score using the top 10 terms (Figure A16d,e,f) between the assigned and true functional terms. The threshold of 10 is chosen based on the observation that most clusters have fewer than 10 enriched terms (Figure A16g,h,i). Across all three DREAM networks and diverse cluster sizes, ReCIPE improves upon the unconnected clusters in the function prediction task, yielding more functionally enriched clusters.

To test that the proteins ReCIPE is adding are meaningful and that the gains in performance are not just coming from adding more proteins, we compare ReCIPE with 50 random bootstraps adding a matched number of random proteins. We use settings of linear ratio = 10%, max proteins = 20. For each cluster, we show the average Jaccard similarity of 50 random bootstraps, compared to the similarity from the ReCIPE cluster (Figure A17, top). We also compute a statistical test of these results, using a Wilcoxon signed-rank test of the median position of the ReCIPE score vs. the random background, against a null hypothesis of 0.5 (i.e. ReCIPE clusters are no better or worse than the random distribution). Across all three networks, we find that ReCIPE significantly improves the Jaccard similarity of clusters (Figure A17, bottom).

Before the ReCIPE step, an individual protein appears in at most one cluster. In A18 we show the distribution of cluster membership for each protein after ReCIPE; most proteins still appear in fewer than five clusters.

Table A4: Statistics of DREAM networks.

	**DREAM 1**	**DREAM 2**	**DREAM 3**
**Nodes**	17397	12420	5009
**Edges**	2232405	397309	18424
**Density**	0.0150	0.00515	0.00134
**Avg. clustering coeffecient**	0.325	0.291	0.134
**Avg. degree**	257	64	7

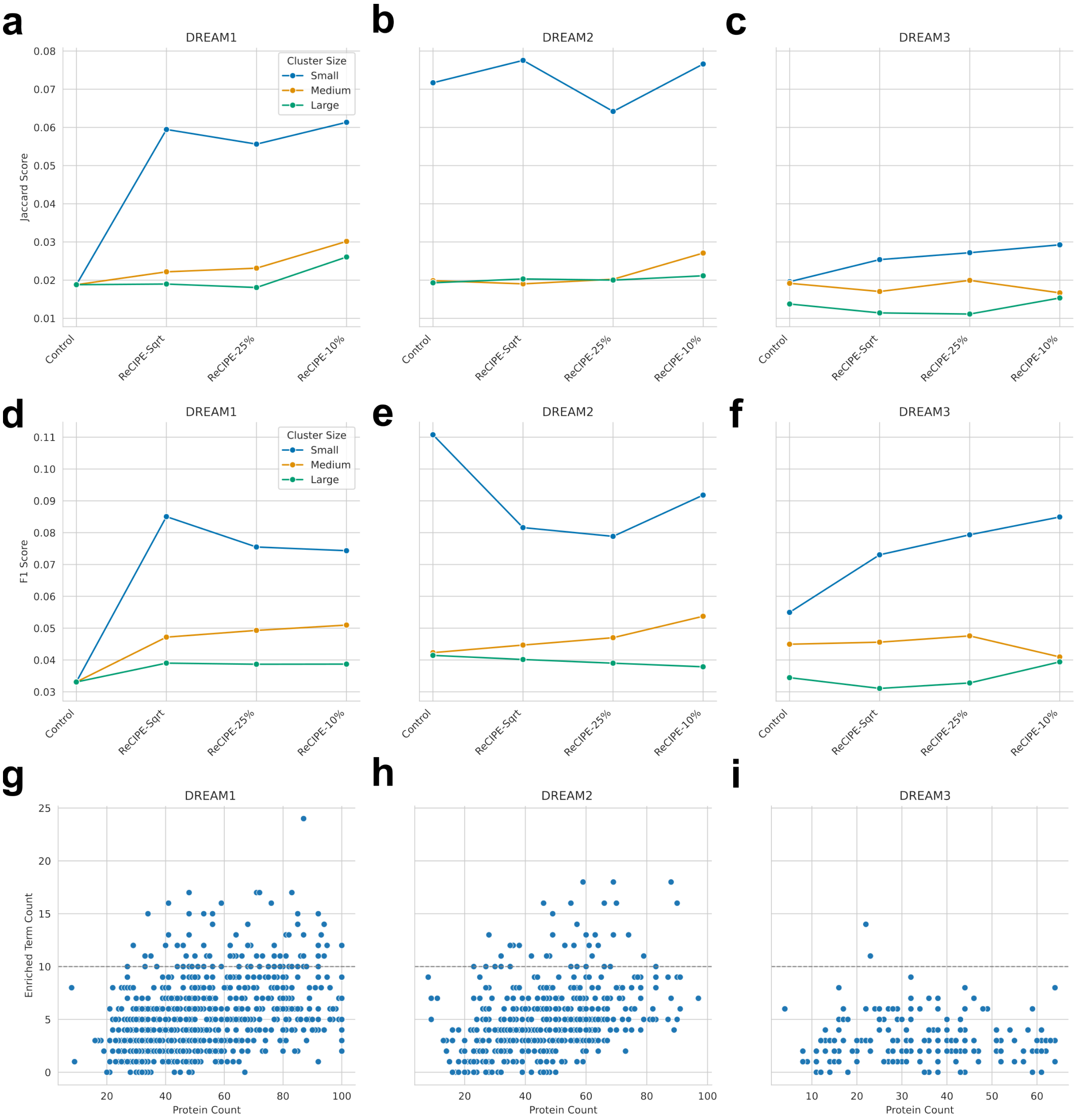

Figure A16: ReCIPE improves percent enrichment of clusters on DREAM networks. (a,b,c) ReCIPE improves Jaccard score of enriched terms on all three DREAM networks. Based on these experiments, we select a linear ratio of 10% to use for our analyses. **(d,e,f)** Using an alternative scoring, where we select the top 10 enriched terms and compute an F1 score, ReCIPE likewise improves on all three networks, except for small clusters in DREAM2. **(g,h,i)** Number of enriched terms for every cluster, plotted by number of proteins in that cluster. We use this to select the threshold of 10 enriched terms (dashed grey line) above, where 92.9%, 94.1% and 99.2% of clusters have fewer than or equal to 10 enriched terms.

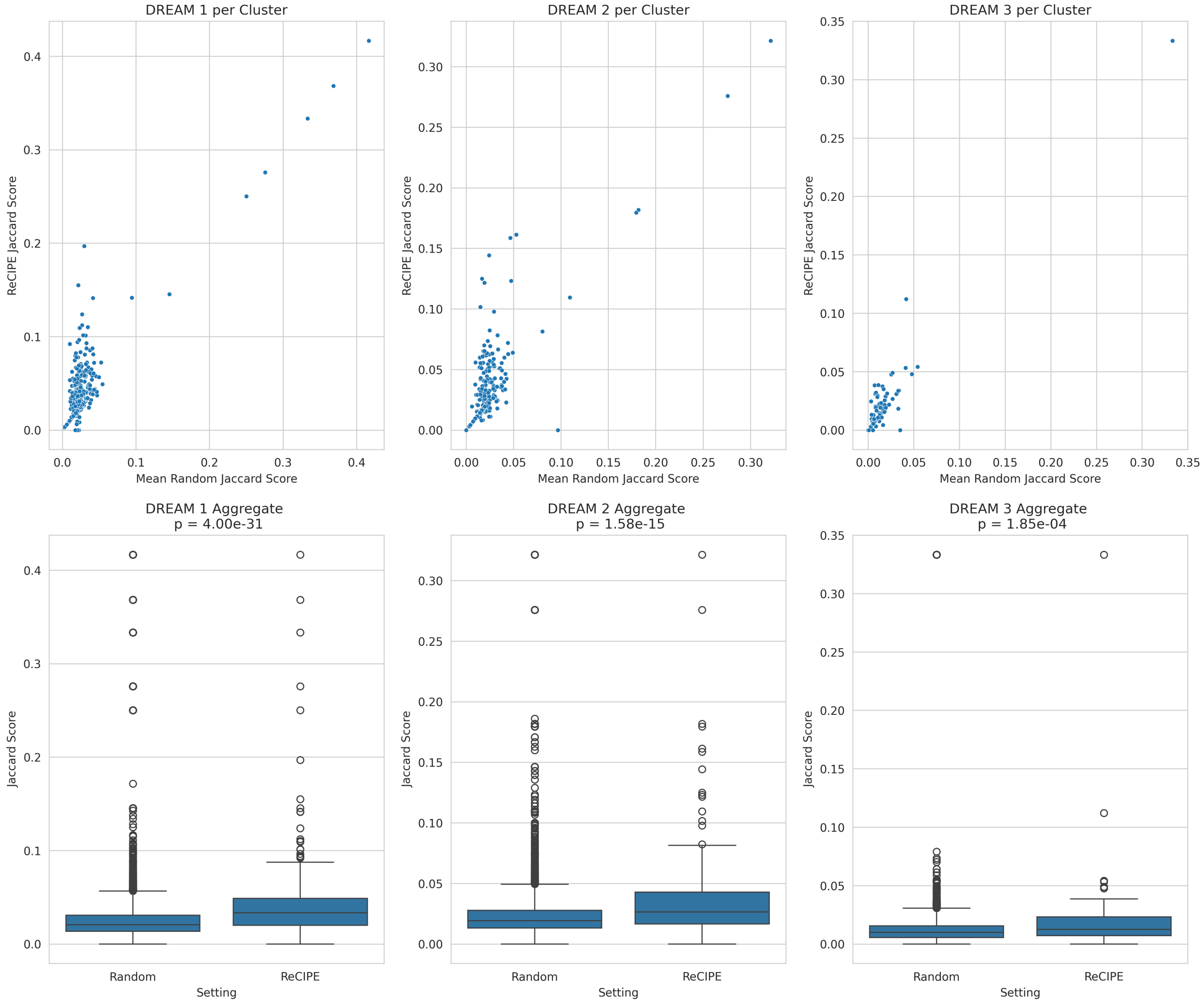

Figure A17: ReCIPE improvement in Jaccard similarity is not driven by larger cluster sizes.

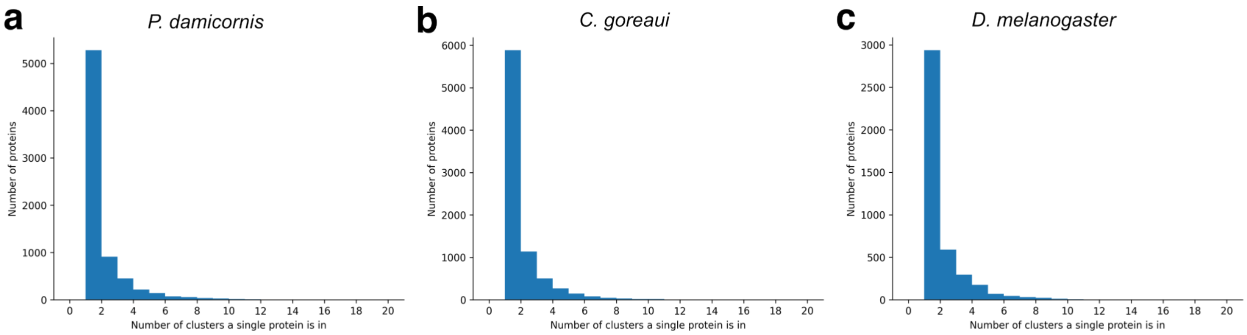

Figure A18: Most proteins are still in a few clusters after ReCIPE. Across all three species evaluated, ReCIPE adds a relatively small number of proteins back to clusters, maintaining them as largely unconnected. Most proteins appear in at most 5 clusters, with only a small handful of proteins appearing more than 8 times.
